# Genomic Data Characterize Reproductive Ecology Patterns in Michigan Invasive Red Swamp Crayfish (*Procambarus clarkii*)

**DOI:** 10.1111/eva.70007

**Published:** 2024-09-15

**Authors:** Nicole E. Adams, Jared J. Homola, Nicholas M. Sard, Lucas R. Nathan, Brian M. Roth, John D. Robinson, Kim T. Scribner

**Affiliations:** ^1^ Department of Fisheries and Wildlife Michigan State University East Lansing Michigan USA; ^2^ U.S. Geological Survey, Wisconsin Cooperative Fishery Research Unit, College of Natural Resources University of Wisconsin‐Stevens Point Stevens Point Wisconsin USA; ^3^ Biological Sciences Department The State University of New York—Oswego Oswego New York USA; ^4^ Michigan Department of Natural Resources Lansing Michigan USA

**Keywords:** effective breeding number, invasive species, kinship‐based approaches, mating behavior, pedigree analysis, *Procambarus clarkii*, reproductive ecology

## Abstract

The establishment and spread of invasive species are directly related to intersexual interactions as dispersal and reproductive success are related to distribution, effective population size, and population growth. Accordingly, populations established by r‐selected species are particularly difficult to suppress or eradicate. One such species, the red swamp crayfish (*Procambarus clarkii*) is established globally at considerable ecological and financial costs to natural and human communities. Here, we develop a single nucleotide polymorphism (SNP) loci panel for *P. clarkii* using restriction‐associated DNA‐sequencing data. We use the SNP panel to successfully genotype 1800 individuals at 930 SNPs in southeastern Michigan, USA. Genotypic data were used to reconstruct pedigrees, which enabled the characterization of *P. clarkii's* mating system and statistical tests for associations among environmental, demographic, and phenotypic predictors and adult reproductive success estimates. We identified juvenile cohorts using genotype‐based pedigrees, body size, and sampling timing, which elucidated the breeding phenology of multiple introduced populations. We report a high prevalence of multiple paternity in each surveyed waterbody, indicating polyandry in this species. We highlight the use of newly developed rapid genomic assessment tools for monitoring population reproductive responses, effective population sizes, and dispersal during ongoing control efforts.

## 1 Introduction

The number of new biological invasions globally has increased dramatically over the last 200 years (Seebens et al. 2017, 2018). Over 6500 non‐native species are established in the United States alone (Tam et al. 2021). Over 450 of those non‐native species are native to parts of North America but have moved outside of their historical range (National Park Service 2022). Not all non‐native species become invasive, but there are multifaceted and wide‐reaching economic and ecological costs for those that do (Haubrock et al. 2021; Fantle‐Lepczyk et al. 2022); costs that have increased exponentially through time (Haubrock et al. 2021). Costs include disruption of ecosystem services, harm to native species, agricultural damage, management services, and threats to human health (Pyšek and Richardson 2010; Gallardo et al. 2016). Since 1960, the combined costs of invasive species have been estimated to be US$140.2 billion in Europe (Haubrock et al. 2021) and US$4.52 trillion in the United States (Fantle‐Lepczyk et al. 2022).

Establishment and subsequent spread of successful invaders are often associated with the species' reproductive characteristics (Sakai et al. 2001). For example, clonal and asexual reproductive strategies can increase abundance in the invaded range, driving postcolonization dispersal (Bazin et al. 2014). For sexually reproducing species, polygamous and promiscuous mating systems can also overcome low founding population sizes and uneven sex ratios (Pannell 2015). Females that mate with multiple males can increase the diversity among offspring and increase the effective population size (*N*_e_), the size of an idealized population that undergoes evolutionary pressures the same as the focal population (Wright 1931), compared to monogamous species (Sugg and Chesser 1994; Pearse and Anderson 2009). Females of some species can store sperm, facilitating reproduction with multiple mates while potentially discriminating sperm from related versus unrelated males (Bretman, Newcombe, and Tregenza 2009). Thus, polygamy within a brood, storage of past mate gametes, and multiple broods in a season can increase genetic and phenotypic diversity and decrease rates of inbreeding at invasion fronts, potentially contributing to rapid population growth and dispersal (Sakai et al. 2001).

Information pertaining to the reproductive biology of an invasive species can inform management decisions, potentially leading to more effective control efforts (e.g., the eradication of *Cochliomyia hominivorax*, the New World screwworm, in the Americas; Klassen and Curtis 2005). Understanding mate choice preferences, for example, such as larger females mating with more males or having greater reproductive success, could lead to targeting larger females for removal during control efforts (e.g., Green and Grosholz 2021; but see Evangelista, Britton, and Cucherousset 2015). In addition, estimating *N*_e_ can help understand how genetic drift (e.g., associated with low abundance) and natural selection (e.g., associated with novel environments) influence population levels of genetic diversity and potential adaptation to novel environments.

Standard field methods to monitor reproductive and demographic features of invasive populations are often labor‐intensive, requiring marking and repeated captures of the same individuals, which impedes detailed understanding of how a species' reproductive biology affects the invasion front. Alternatively, reconstructed genetic pedigrees of individuals sampled in a single capture event can be used to estimate many population parameters (e.g., Bravington, Grewe, and Davies 2016; Marcy‐Quay et al. 2020; Sharma et al. 2022). For instance, genetic pedigrees can characterize aspects of mating systems such as the rates of multiple paternity, reproductive success distributions, and dispersion patterns among siblings (e.g., Walker, Porter, and Avise 2002; Yue et al. 2010; Gibson et al. 2020; Prakash et al. 2022). In addition, information in reconstructed pedigrees can be used to estimate measures associated with population size (e.g., Ruzzante et al. 2019; Sard et al. 2021), population growth rates (e.g., Anderson et al. 2015; Evans et al. 2016), and dispersal (Ford, Murdoch, and Hughes 2015; Baetscher et al. 2019), as well as measures of effective size at cohort and population scales (Wang 2009, 2016; Waples and Do 2010; Waples, Antao, and Luikart 2014).

The effective number of breeding individuals contributing to a reproduction event (*N*_b_) can be estimated using samples from a single cohort (Waples, Antao, and Luikart 2014). Estimates of the number of effective breeders can give insight into the census size of successfully breeding adults in established populations. Estimates of the number of successfully breeding adults (*N*_s_) can also be quantified using recently developed pedigree‐based techniques (Sard et al. 2021). Estimates of *N*_b_ based on pedigree reconstruction or asymptotic numbers of breeding adults from pedigree accumulation analyses (Sard et al. 2021) can inform managers whether management prescriptions were effective in reducing population abundance (e.g. Athrey et al. 2012; Zalewski et al. 2016). However, the quality of such inferences rests on the quality and quantity of loci genotyped.

Targeted genomic sequencing approaches, such as restriction site–associated DNA (RAD) capture (Ali et al. 2016), enable hundreds of individuals to be genotyped at a genome‐wide sample of loci with low genotyping error rates. As a result, pedigrees for invasive species sampled in the field can be reconstructed more accurately and the quality of associated ecological and evolutionary inferences improves. Genomic resources are increasingly being created and applied to study invasive species (Sard et al. 2020; Vu et al. 2023). However, more resources could support management decision‐making.

The red swamp crayfish (RSC; *Procambarus clarkii*) is a prolific and globally introduced invasive species (Gherardi et al. 2011; Loureiro et al. 2015). *Procambarus clarkii* is native to the southcentral United States and northern Mexico but is established on every continent except for Australia and Antarctica through aquaculture, bait trade, the pet trade, or biological supplies for classrooms (Hobbs, Jass, and Huner 1989; Chucholl 2013; Loureiro et al. 2015; Smith et al. 2018; Oficialdegui, Sánchez, and Clavero 2020). Introduced *P. clarkii* are demonstrated to have strong negative effects on native species (Souty‐Grosset et al. 2016) and ecosystem integrity (Tricarico et al. 2010). Adverse effects are particularly notable for aquatic taxa, specifically for amphibians, fish, invertebrates, macrophytes, and native crayfish (Rodríguez, Bécares, and Fernández‐Aláez 2003; Gherardi and Acquistapace 2007; Matsuzaki et al. 2009; Twardochleb, Olden, and Larson 2013; Souty‐Grosset et al. 2016). *Procambarus clarkii* is also known to be a vector for a pathogen that causes crayfish plague, which causes native crayfish mass mortality in species without immunity (i.e., outside North America) (Mrugała et al. 2017; Martín‐Torrijos et al. 2018).

Established invasive populations of *P. clarkii* were found in 2017 near Detroit, Michigan, United States (Smith et al. 2018). Following the first documentation, a collaborative response effort began to limit spread and eradicate populations (Budnick et al. 2022). Control efforts have included trapping and removal (Budnick et al. 2022), burrow excavations, carbon dioxide treatments (Smerud et al. 2022), and pesticide treatments (unpublished data). Globally, invasive *P. clarkii* populations have been treated with pyrethroid insecticides in attempts to reduce population size and control spread (e.g., Cecchinelli et al. 2012; Wu et al. 2012; Lidova et al. 2019). In July of 2021, the Michigan Department of Natural Resources (DNR) treated one of the Detroit waterbodies with a pyrethroid insecticide to test the efficacy of chemical control in these newly found invasive populations. By using genomic data, we can further understand the effectiveness of this chemical control effort in reducing the population, especially compared to the other untreated populations.

Differences in reproductive patterns from the native to invaded ranges are being revealed. Importantly, patterns may differ substantially among the multiple, independently introduced populations (Sard et al. 2023) of *P. clarkii*. Specifically, the timing of breeding, operational sex ratios of successful reproducing adults, interindividual variation in reproductive success, and population recruitment levels can vary associated with different invading source populations (genetic effects) or plastically due to local environmental variables (Sommer 1984; Alcorlo, Geiger, and Otero 2008; Chucholl 2011). For example, in some cooler climates, *P. clarkii* have one or two mating periods (Suko 1958), but in warm climates, *P. clarkii* is demonstrated to breed year‐round (Penn 1943; Huner and Barr 1991), suggesting that the number of cohorts per year varies with differences in climate. The egg incubation period also depends on environmental conditions and can range from 2 to 3 weeks during warmer summer temperatures to 2 to 3 months at late winter colder temperatures (Suko 1956, 1958; Huner and Barr 1991). Young hatch from eggs in mid‐summer and often remain in a burrow with the female for 6 to 12 weeks (Suko 1958; Huner and Barr 1991). Under ideal conditions, a generation can be produced in 4.5 months with some populations having 1.5 to 2 generations per year (Suko 1958; Huner and Barr 1991). Little is known about reproductive patterns within the Detroit invasion. *Procambarus clarkii* populations in Michigan are from multiple independent introductions, lie well north of their native range, and experience comparatively shorter summers and longer, colder winters. Thus, previous research (e.g., Huner and Barr 1991) could have limited applicability for *P. clarkii* reproductive habits in these areas. Understanding the reproductive dynamics in Michigan may help managers better understand the dynamics in other cooler climate invasive populations globally. However, there is still a critical need to understand local dynamics to tailor management practices that target species' attributes that can contribute to limiting local recruitment and range expansion.

Genomic tools are not always available for nonmodel organisms, which is often the case for invasive species. Therefore, there is a need to develop genetic tools for invasive species. Informative genetic marker panels can facilitate analyses that are standardized and cost‐efficient. In this study, we first develop a set of high‐quality baits to target polymorphic SNP loci as a critical public resource for genetic monitoring of *P. clarkii* in Michigan and globally. Project objectives were to develop and use SNP loci and pedigree analyses to characterize the reproductive biology of *P. clarkii*, specifically the number and timing of juvenile cohort production, estimates of the effective number of breeding adults (including a comparison before and after chemical eradication efforts in one population), and the incidence of multiple paternity in five invasive *P. clarkii* populations in the metropolitan area of Detroit, Michigan, USA. We then discuss management implications, such as the timing of control treatments, and highlight the major findings from population genomic data into invasive species control efforts.

## 2 Methods

### 2.1 Sample Collection and Processing

**FIGURE 1:**
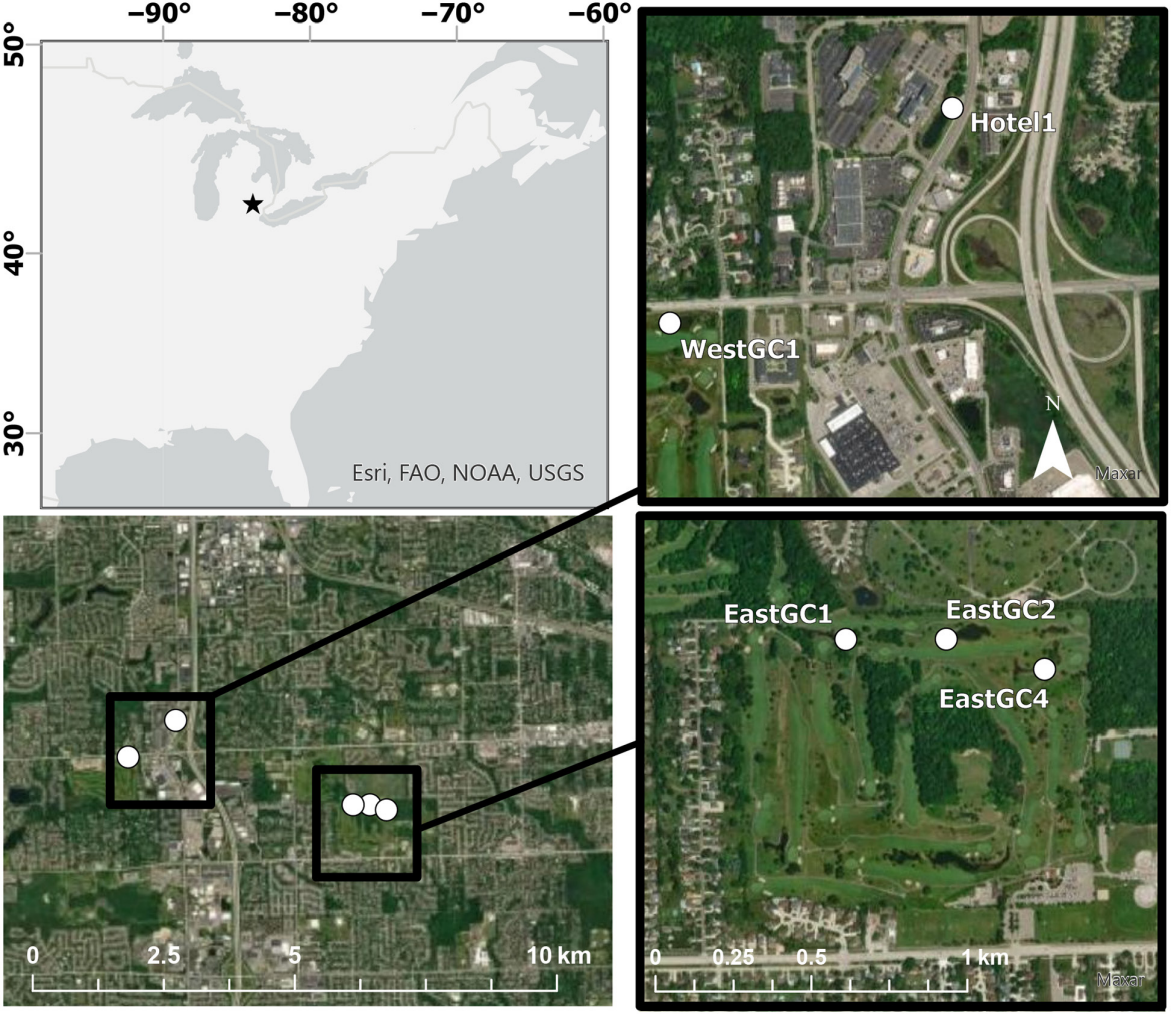
Map of waterbodies sampled for invasive *Procambarus clarkii* in southeastern Michigan, USA. The waterbodies are shown as two geographic groups in black squares on the bottom left map and shown at a closer scale in the maps on the right.

*Procambarus clarkii* were collected from five waterbodies in southeast Michigan, USA, between May and October in 3 consecutive years (2019–2021; Figure 1, Table S1). All five waterbodies are thought to be from one founding source (unpublished data). Four waterbodies were located on two golf courses (East Golf Course 2 (EastGC2), East Golf Course 1 (EastGC1), East Golf Course 4 (EastGC4), and West Golf Course 1 (WestGC1)) and one was a retention pond (Hotel 1 (Hotel1)). Samples were collected as part of ongoing removal efforts by the Michigan DNR and Michigan State University (MSU). Gee‐style minnow traps baited with dog food and unbaited artificial refuge‐style traps were checked regularly (Monday–Friday). Adults and juveniles were opportunistically collected from either trap type. However, the minnow traps are demonstrated to attract larger crayfish, and the artificial refuge‐style traps attract smaller crayfish, depending on the time of year (Budnick et al. 2022). Traps were deployed equidistantly at a density of one trap per 5 m of shoreline, so the total number of traps was not equal across all ponds. Berried females (females with attached eggs and hatchlings) were specifically targeted by excavating burrows. For further fieldwork details, see Budnick et al. (2024). From 2019 to 2021, there were 1018 total surveys and 40,030 traps deployed across these five waterbodies. Over those 3 years, a total of 94,265 *P. clarkii* were removed, ranging from 8544 removed from EastGC2 to 24,670 removed from EastGC1. At Hotel1, 10,826 traps were deployed, which removed 19,160 crayfish from 2019 to 2021. Crayfish at Hotel1 were collected opportunistically before and after a pyrethroid treatment (ExciteR; Zoëcon, Schaumburg, IL, USA) in July 2021. No other waterbodies in this study were treated with ExciteR during our sampling period.

A subset of the captured *P. clarkii* were placed in 95% ethanol for genetic analyses. Early efforts resulting in poor DNA quantity and quality suggested that tissue degraded when left in the original ethanol, likely due to higher water content stored in the body. Therefore, ethanol was changed within the first 5–7 days after collection, then again once every 2 weeks until the samples could be processed to preserve tissues for DNA extraction and genotyping. For every individual except for eggs and hatchlings, we recorded capture date, sex, and carapace length measured from the tip of the rostrum to the posterior end of the carapace with a digital caliper. We followed the descriptions in Huner and Barr (1991) to distinguish sexes and used 20 mm carapace length to distinguish juveniles from adults in the field based on inspection of previous field data collected in Michigan (Budnick et al. 2022). For each site, we had a target sample size of ≥30 adults.

### 2.2 DNA Extraction and Quantification

Gill tissue was taken from each adult and juvenile *P. clarkii* for DNA extraction. For the eggs and hatchlings, whole organisms were used for the DNA extractions. Genomic DNA was extracted from tissue using either a spin column‐based approach (DNeasy Blood and Tissue kit; Qiagen, Hilden, Germany), following the manufacturer's protocols, or a bead‐based protocol in 96‐well plates, following methods in Ali et al. (2016). Briefly, the Ali et al. (2016) protocol involved air‐dried tissue being digested overnight at 56°C in a mix of Lifton's buffer, proteinase K, and 1 M DTT. We then added magnetic beads (Sera‐Mag SpeedBeads; Cytiva, Marlborough, MA, USA) in a hybridization buffer (DTT, NaCl, PEG 8000, and water) to isolate the DNA following Rohland and Reich (2012). This was followed by two 80% ethanol washes using a plate magnet before genomic DNA was eluted in 60 μL of DNA Suspension Buffer (Teknova, Hollister, CA). Initial DNA quantification was performed using a spectrophotometer (NanoDrop 1000; Thermo Fisher Scientific, Waltham, MA, USA) to ensure DNA concentrations were within the effective range of subsequent intercalating fluorophore‐based quantification (Quant‐iT PicoGreen dsDNA Assay Kit; Invitrogen, Waltham, MA, USA) that was measured using a quantitative PCR instrument (QuantStudio 6 Real‐Time PCR System; Thermo Fisher Scientific). DNA concentrations were then standardized across samples to <80 ng/μL prior to restriction site‐associated DNA (RAD) library construction.

### 2.3 Library Preparation and Sequencing

RAD capture libraries were prepared using the BestRAD protocol (Ali et al. 2016). The RAD capture approach improves upon traditional RAD methods by decreasing the amount of PCR clones, which can lead to incorrect genotype calls (Hohenlohe et al. 2013; Andrews et al. 2014), by employing physical rather than PCR enrichment of RAD tags thereby recovering more unique RAD fragments (Ali et al. 2016). Overall, RAD capture produces consistently higher concentration and quality libraries, is more cost‐effective, and is scalable to thousands of samples (Ali et al. 2016), making the method particularly useful for pedigree analyses. Finally, by identifying and creating the RAD capture bait set, this standardized, cost‐effective resource can be used for genetic monitoring of *P. clarkii* globally. Briefly, genomic DNA was digested with *SbfI* restriction enzyme (New England Biolabs, Ipswich, MA, USA). BestRAD adapters (New England Biolabs) were ligated to the cut ends using T4 Ligase (New England Biolabs). Pooled samples were eluted in 135 μL of low Tris‐EDTA (TE) buffer and then sheared with a sonicator (Covaris M220; Woburn, MA, USA) for an average target fragment length of 325 base pairs (bp). Barcoded, sheared DNA was isolated with streptavidin beads (Dynabeads M‐280; Invitrogen), then libraries were prepared following NEBNext Ultra (or Ultra II for plates 13–24) Library prep kit for Illumina (New England Biolabs). Libraries were dual indexed using NEB Dual Index Sets 1–4 and amplified for 12 cycles, followed by a magnetic bead‐based (Ampure XP bead; Beckman Coulter, Brea, CA, USA) cleanup. Libraries were quantified using a fluorometer (Qubit, Thermo Fisher Scientific), and the quality was assessed using microfluidic automated electrophoresis (Bioanalyzer; Agilent, Santa Clara, CA, USA).

To include all libraries and minimize the number of reactions, one to four capture reactions, each containing 6 to 12 pooled libraries, were carried out using the MyBaits Version 4.01 protocol (https://arborbiosci.com/wp‐content/uploads/2018/04/myBaits‐Manual‐v4.pdf). For details on the RNA bait development, see Appendix S1. RNA baits were allowed to hybridize to *P. clarkii* BestRAD libraries for 16 h at 65°C and then washed with Wash Buffer X at between 65 and 67°C. Washed capture reactions were amplified for 11 cycles using KAPA Library Amplification Kit for Illumina (KAPA Biosystems, Wilmington, MA, USA) and quantified using a Qubit. Quality was assessed using an automated electrophoresis (TapeStation; Aligent). The four capture libraries were then pooled into one sequencing library that included equal DNA quantities from each of the capture libraries, which was then assessed with Qubit and a TapeStation. The RAD capture library was then sequenced on one lane of Illumina's NovaSeq 6000 as 150 bp paired‐end reads at the Research Technology Support Facility (RTSF) Genomics Core at MSU. We created 36 total libraries for two independent sequencing runs. The first 12 were sequenced in 2021 and the remaining 24 were sequenced in 2022. Base calling was done by Illumina Real Time Analysis (RTA) v3.4.4, and output of RTA was demultiplexed and converted to FastQ format with Illumina Bcl2fastq v2.20.0 by the RTSF. Additional samples of *P. clarkii* for a subsequent project were simultaneously prepared and sequenced, which is why there were 36 plates of individual libraries. Individual sequences after demultiplexing libraries are available on the NCBI sequence read archive (Bioproject PRJNA1148680).

### 2.4 Bioinformatic Processing

RAD capture sequence reads in the forward and reverse files for each library were exchanged whenever the barcode was found at the start of read 2 using a previously published perl script (bRAD_flip_trim.pl; originally developed by Paul Hohenlohe, University of Idaho, and modified by Brian Hand and Seth Smith, University of Montana). Libraries were demultiplexed using process_radtags, and PCR duplicates were removed using clone_filter in Stacks v. 2.59 (Catchen et al. 2013). Illumina adapter sequences and reads shorter than 50 bp were removed, and reads were trimmed if the mean base quality dropped below Q15 using a sliding window of four bases using Trimmomatic v. 0.39 (Bolger, Lohse, and Usadel 2014). Reads were mapped to the *P. clarkii* reference genome (GCA_020424385.2; Xu et al. 2021) using BWA‐MEM v. 0.7.17 (Li and Durbin 2010; Li 2013). Mapped reads were sorted and indexed, then reads with mapping qualities <20, and those not mapped in proper pairs were filtered out using SAMtools v. 1.9 (Li et al. 2009). Genotypes were then called using the gstacks module of Stacks v. 2.4 (Catchen et al. 2013; Rochette, Rivera‐Colón, and Catchen 2019). Read depth was evaluated with pybedtools v. 0.8.1 (Quinlan and Hall 2010; Dale, Pedersen, and Quinlan 2011).

### 2.5 Site Filtering

To remove the most poorly sequenced samples and loci, we first filtered out SNPs with quality scores of < 20, minor allele counts of < 3 (allele count defined as the number of times that allele appears over all individuals at that SNP position), > 99% missing data, and read counts of 2 or fewer. We then removed individuals with >99% missing data, essentially samples that failed to sequence. For the remaining samples and loci, we did a subsequent round of filtering that removed genotype calls based on fewer than seven reads within an individual and kept only those loci genotyped in at least 75% of individuals. Next, we removed sites with observed heterozygosity >0.6 and allele balance values >0.6 and <0.4 (McKinney et al. 2017; Weise et al. 2022). For pedigree reconstruction, we kept only one SNP per RAD tag. Specifically, we retained the locus with the lowest frequency of missing data and the highest minor allele frequency from each RAD tag. If two or more SNPs met all the criteria, the first SNP was selected. These variant filtering steps are similar to Weise et al. (2022).

For analyses involving juveniles (*N*_b_, *N*_s_, coancestry, and reproductive success), we additionally removed individuals with >50% missing loci and kept loci with a minimum of 20 reads and a minimum minor allele frequency (maf) of 0.005. For multiple paternity analyses, we used berried females, their offspring (fertilized eggs and hatchlings), and adult males from the same waterbody collected over the course of the study. For this dataset, we removed individuals with >75% missing data to keep as many berried females as possible and retained loci with a minimum of 20 reads per individual and a maf of 0.3. Differences in filtering steps were due to differences in data quality in the two sets of samples.

### 2.6 Defining Juvenile Cohorts

To determine full‐ and half‐sibling relationships (and unrelated individuals), we constructed pedigrees based on juveniles using the program Colony (Jones and Wang 2010). We first converted VCF files to Colony‐formatted data using code modified from existing scripts (vcf_colony.R, colonydat_create.R; Sard et al. 2021; Weise et al. 2022). Input parameters for Colony included dioecious species type, polygamous mating system, allowed for inbreeding, unknown allele frequencies, and sibship scaling, but no prior sibship was reported. The initial genotyping error rate was set at 0.01 and the initial random error was set at 0.001—Colony estimates error rates during the analysis (Wang 2004). Sibling relationships were estimated with the full‐likelihood approach in Colony v 2.0.6.7 (Jones and Wang 2010) using a long run with medium‐likelihood precision. Juvenile cohorts were determined for each waterbody based on body size–frequency distributions, date of capture, and inferred sibling relationships. We also calculated pairwise measures of interindividual relatedness among juveniles collected from each waterbody (not cohorts) based on the KING inference (Manichaikul et al. 2010) using VCFtools (Danecek et al. 2011). We only tested for significant differences in the KING relatedness between groups that had low *F*_ST_ (< 0.1, data not shown), which was EastGC2 versus EastGC4 and between Hotel1 cohorts. The median KING relatedness value for each group was calculated and the ratio between pairwise group comparisons was used as a test statistic. Each test statistic was compared to a null distribution generated by a randomization procedure that was run 1000 times. The ratio from the empirical data was compared to the randomized distribution to test for statistical significance.

### 2.7 Estimating Breeding Adult Number and Effective Number of Breeding Adults

Within each juvenile cohort and sampling location, we calculated estimates of the effective number of breeding adults (*N*_b_) using two methods. The first estimate (*N*_b_LD_) used a linkage disequilibrium model assuming random mating implemented in NeEstimator v 2.1 (Do et al. 2014). To make our data format compatible with NeEstimator, we first used PGDspider v 2.1.1.5 (Lischer and Excoffier 2012) to convert each VCF to a GenePop file. Instead of filtering out multiple SNPs on a single RAD tag, we supplied a chromosome map to NeEstimator following (Waples, Larson, and Waples 2016) to avoid intrachromosome (physically linked) comparisons. The second approach was based on the frequency of sibling relationships identified using Colony reconstructed pedigrees (*N*_b_Wang_; Wang and Santure 2009).

We report the estimate of the number of reconstructed parental genotypes based on the offspring pedigrees without adjustment (*N*_s_). In addition, the asymptotic number of successfully breeding adults, contributing reproductively to each location and cohort ($\hat{N_{s}}$), was calculated using a parentage accumulation curve approach (*N*_s__calc.R; Sard et al. 2021; Weise et al. 2022). The asymptotic value, $\hat{N_{s}}$, was based on the Chao estimate of the total number of parental genotypes contributing to each cohort calculated using the specpool function in the R package vegan (Oksanen et al. 2022). We estimated the mean and variance in reproductive success (RS) for contributing adults based on the number of offspring assigned to each individual in the reconstructed pedigree from Colony. This reproductive success metric is similar to $\underline{k}$ except our dataset did not include adults without offspring. We randomly subsampled the Colony output to 75 based on the number of juveniles in the cohort with the lowest sample size (*N* = 90), Hotel1 cohort1, to make interwaterbody comparisons of reproductive success. We repeated the subsampling 1000 times and then calculated the mean, standard deviation, and 95% confidence interval across replicates. Additionally, coancestry (Θ) was estimated based on the number of half‐ and full‐siblings and unrelated offspring pairs using the reconstructed pedigrees (coancestry.R; Scribner et al. 2022). Analyses of the Hotel1 pond individuals were conducted separately for each cohort and on the combined cohorts to get estimates for the full pond.

### 2.8 Estimates of Multiple Paternity

We used one to six berried females per waterbody to calculate estimates of multiple paternity, which were collected in 2020 (*N* = 10) or 2021 (*N* = 12; Table 2, Table S1). Multiple paternity was defined as offspring from one berried female assigned to more than one inferred male from the Best Config Colony file. We modified the existing vcf_colony function (vcf_colony.R; Weise et al. 2022) to include separate entries for candidate adult females and adult males and modified the colonydat_create function (colonydat_create.R; Weise et al. 2022) to include berried female and offspring dyads when converting VCF files to Colony‐formatted data. Input parameters for Colony were the same as those used with the juvenile dataset but with two additional parameters: 99% probability of including the mother and a 1% probability of including the mate. These parameters were chosen because eggs and hatchlings were taken from the female's abdomen or from the bottom of the container that housed individual berried females giving us high certainty of the offspring's mother. However, we only included 14–72 males per waterbody, which is a small fraction compared to the estimated population sizes giving us low confidence that the other parent was included in our dataset.

We tested for associations between body size (carapace length) of the berried females and the estimated number of mates (reconstructed male parental genotypes represented in sampled offspring) using pedigree data from all waterbodies using generalized linear models. Our global model included the following variables: the number of offspring genotyped per berried female, waterbody, waterbody‐specific catch per unit effort (estimate of population density), and the year the berried females (and offspring) were collected. The Michigan DNR provided estimates of mean catch per unit effort (CPUE), which was calculated from individuals (adults and juveniles) caught in Gee's minnow traps during August in each waterbody (Table S2). We focused our analyses on August CPUE to limit the influence of juvenile recruitment on relative abundance estimates. Models were evaluated with the stepAIC function in the R package MASS (Venables and Ripley 2002) using both backward and forward stepwise searches. Multicollinearity among variables was accounted for within stepAIC. Since previous work explicitly evaluated the number of mates versus berried female body size (Yue et al. 2010; Hamasaki, Tsuboi, and Dan 2022), we specifically plotted that relationship and used a linear model to assess the relationship. We excluded EastGC2 and one berried female (Hotel1‐M14‐20) from the models due to low sample sizes.

## 3 Results

### 3.1 RAD Capture Bait Discovery

Based on 175 *P. clarkii samples*, our RAD discovery library resulted in the genotyping of 245,717 RAD loci containing 1,064,629 SNPs. Over 79% of reads had a single primary alignment to the marbled crayfish (*Procambarus virginalis*) reference genome (Gutekunst et al. 2018). Because the red swamp crayfish genome (GCA_020424385.2; Xu et al. 2021) was not yet available, we mapped reads to the *P. virginalis* reference genome (NCBI Bioproject accession number PRJNA356499; Gutekunst et al. (2018)) (Appendix S1). Effective per‐sample read depths per RAD locus averaged 13.7× and ranged from 1.2 to 59.3× per individual. A total of 7660 capture baits that passed all quality control filters were developed to genotype 2620 RAD loci (200 with two‐tiled baits and 2420 with three‐tiled baits) to be used on the remaining *P. clarkii samples*.

A total of 7128 baits (93%) were mapped to the *P. clarkii* genome (Figure S1). We quantified the number of baits that generated genotypes using BEDTools intersect (Quinlan and Hall 2010) to identify overlap between targeted RAD loci and the VCF file produced by gstacks. We recovered genotypes from 6642 (93%) of the 7128 mapped baits and 87% of the 7660 total baits.

### 3.2 Sequencing Results and Genotyping Using RAD Capture Baits

The mean PCR duplication rate was 66%, and we retained a total of 575,777,966 paired reads after removing duplicates. On average, 93% of read pairs were maintained after read quality trimming, and 95% of sequence reads were successfully mapped to the *P. clarkii* reference genome. Genotyping resulted in 200,062 unfiltered RAD loci and 1,012,163 unfiltered variant sites. Over 92% of reads had a single primary alignment to the *P. clarkii reference genome*. Effective per‐sample read depths per RAD locus averaged 28.0× (SD = 23.3×) and ranged from 1.0× to 209.5×. The filtered data contained just over 1340 unique RAD tags.

### 3.3 Defining Juvenile Cohorts and Relatedness

**FIGURE 2:**
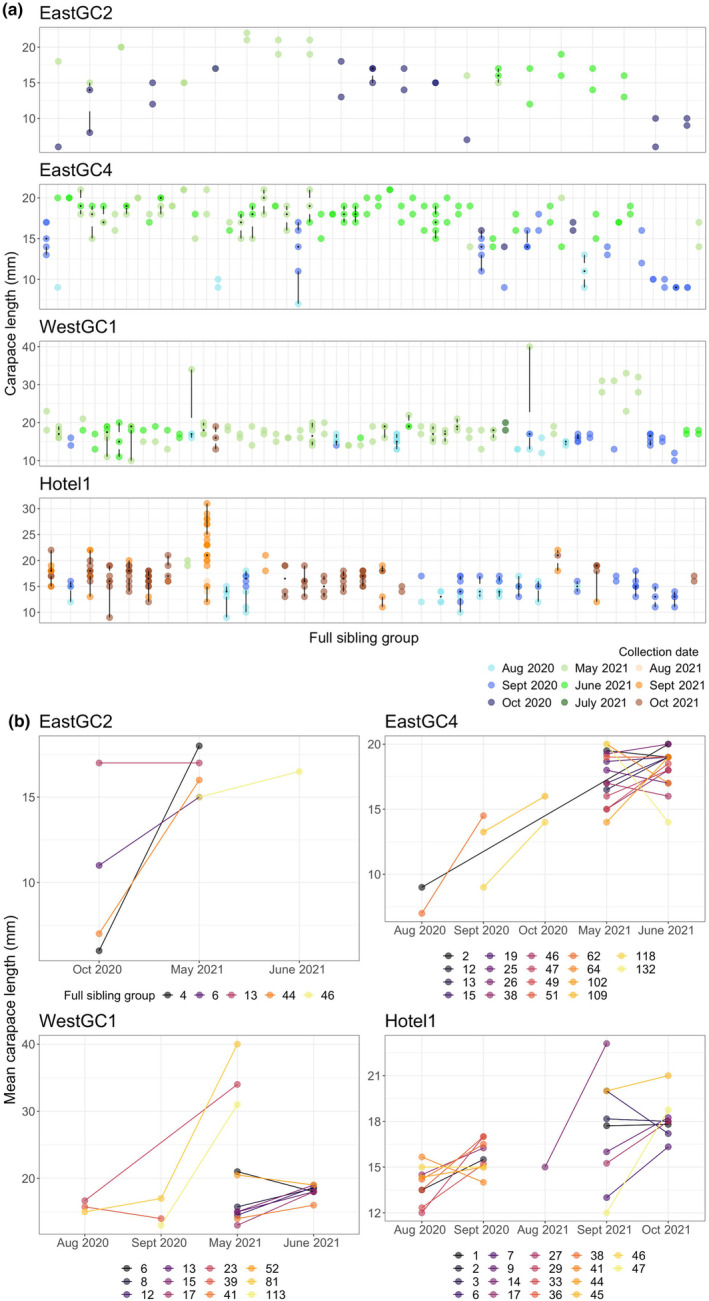
(a) Modified boxplots of carapace length of inferred full‐sibling relationships among *Procambarus clarkii* juveniles collected from four sites (East Golf Course 2, East Golf Course 4, West Golf Course 1, and Hotel1) used to determine the number of juvenile cohorts present at each site. Black dots show the median carapace length, the white gap illustrates the interquartile range, and lines extend from the lower and upper quartiles to the minimum and maximum carapace length for each inferred family. Colored points show the month the individual was collected. Full‐sibling groups with a single individual were removed from the plots. (b) Mean carapace length (mm) of inferred full‐sibling juvenile groups that were collected over more than 1 month. Sibling relationships were estimated with Colony based on 930 single nucleotide polymorphisms (SNPs).

**FIGURE 3:**
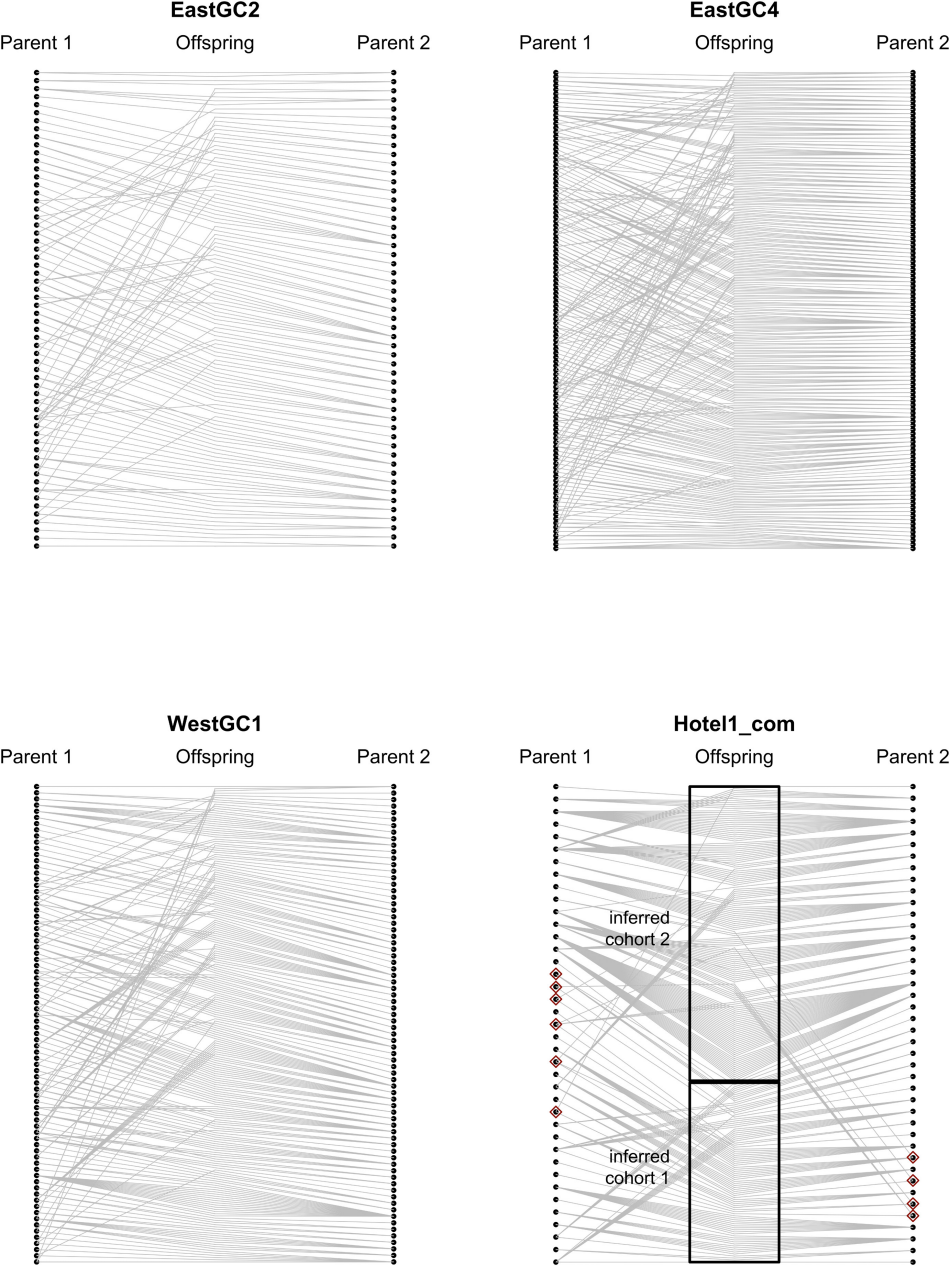
Visualization of reconstructed *Procambarus clarkii* pedigrees based on juveniles in four waterbodies (East Golf Course 2, East Golf Course 4, West Golf Course 1, and Hotel1). Hotel1 juveniles are split into cohorts 1 (co1) and 2 (co2). The center points represent genotyped juveniles, and the lines indicate relationships between inferred parent 1 and inferred parent 2. Inferred Hotel1 parents that produced juveniles in both cohorts are indicated with red diamonds.

We defined juvenile cohorts based on the size of collection grouping juveniles collected in the fall of 2020 and the spring of 2021 together. Juveniles with smaller carapace lengths were found in the fall compared to the spring and summer (Figure 2). Based on a Dunn test of carapace length versus collection time, the most significant tests after a Bonferroni correction were between juveniles collected in Fall 2020 and those collected in Spring 2021 (May 2021–September 2020 *Z* = 11.3, *p* < 2 × 10^−16^; May 2021–October 2020 *Z* = 10.8, *p* < 2 × 10^−16^). Full‐sibling groups were characterized by smaller juveniles in the fall and larger siblings in the spring. For example, three of four sibling groups from waterbody EastGC2 had larger individuals in May 2021 compared to October 2020 (Figure 2b). A second cohort of smaller individuals was collected in summer and fall of 2021 in both WestGC1 and Hotel1. There were only five individuals in the second cohort in WestGC1, so they were removed from further analyses. Visualization of the family structure across the inferred cohorts can be found in Figure 3, in which genetically inferred parents are to the right and left of the genotyped juvenile in the middle. An inferred parent that gave rise to multiple juveniles has many gray lines connecting them to their offspring. In the panel for Hotel1, we can see inferred parents that contributed offspring in multiple cohorts (Figure 3).

The distribution of pairwise interindividual relatedness estimates trended toward significantly different among juveniles sampled between EastGC2 and EastGC4 (*p* = 0.051, two‐tailed test), as well as between Hotel1's cohort1 and cohort2 (*p* = 0.026, two‐tailed test; Figure S2). The difference between cohorts at Hotel1 may be explained by differences in sample sizes (cohort1 = 8010 pairwise comparisons, cohort2 = 21,462). Mean coancestry estimates among juveniles ranged from 0.002 in EastGC4 to 0.021 in the second Hotel1 cohort (Table 1).

**TABLE 1:** Summary of reproductive statistics estimated for juvenile red swamp crayfish (*Procambarus clarkii*) for four sites (East Golf Course 2, East Golf Course 4, Meadowbrook 1, and Hotel1).

	EastGC2	EastGC4	WestGC1	Hotel1 (cohort 1)	Hotel1 (cohort 2)	Hotel1 (combined)
Cohort dates	Fall2020–Spring2021	Fall2020–Spring2021	Fall2020–Spring2021	Fall2020–Spring2021	Summer2021–Fall2021	Fall2020–Fall2021
Sample size	118	294	218^a^	90	147	237^a^
*N*_b_LD_	172.2 (105.3–390.7)	197.3 (154.7–261.8)	66.7 (44.8–105.3)	27.0 (20.1–37.3)	17.6 (15.3–20.3)	33.7 (29.3–38.8)
*N*_b_Wang_	145 (114–191)	253 (211–302)	121 (96–157)	33 (21–55)	24 (14–43)	49 (34–73)
*N*_s_	113	230	152	46	41	81
$\hat{N_{s}}$	124.9 ± 5.6	243.2 ± 5.5	169.9 ± 7.4	74.6 ± 19.6	45.1 ± 4.0	93.0 ± 7.6
RS (95% CI)	1.62 ± 0.055	1.32 ± 0.053	1.62 ± 0.085	3.50 ± 0.148	4.25 ± 0.273	2.58 ± 0.155
	(1.52–1.74)	(1.23–1.44)	(1.47–1.81)	(3.26–3.85)	(3.75–4.84)	(2.31–2.94)
Θ	0.004	0.002	0.004	0.015	0.021	0.010

*Note:* Definition of cohorts by collection dates, sample sizes of red swamp crayfish juveniles used in analyses, and the estimated effective number of breeders estimated using NeEstimator (*N*_b_LD_) and Colony (*N*_b_Wang_). *N*_b_LD_ estimates are followed by a jackknife confidence interval and *N*_b_Wang_ estimates are followed by 95% confidence intervals. Estimates of reconstructed parental genotypes based on the pedigrees without adjustments (*N*_s_) and estimates of the asymptotic number ($\hat{N_{s}}$) of adults based on the Chao estimate with a standard error, estimated mean rarefied reproductive success (RS) of contributing adults with aSD and 95% confidence interval, and mean offspring coancestry (Θ) are also included. Analyses are based on 930 genetic markers (single nucleotide polymorphisms, SNPs), except for *N*_b_LD_ which was based on 2262 SNPs.

^a^ The total number of samples genotyped was 249 for WGC1 and 241 for Hotel1, but samples were removed (WGC1 *N* = 30 and Hotel1 *N* = 4) because they had a carapace length >20 mm or were in a second cohort collected from WGC1 that did not have a large enough sample size to analyze.

### 3.4 Estimates of the Number of Contributing Adults

Reconstructed pedigrees identifying sibship relationships and inferred parents for sampled juveniles (Figure 3) in each waterbody revealed a broad range in reproductive success among inferred parents. Most individuals contributed multiple offspring to a cohort (Table 1). The average number of offspring per parent ranged from 2.09 ± 1.06 SD in EastGC2 to 7.17 ± 6.58 SD in the second Hotel1 cohort, which was after the control treatment (Table 1). In the most extreme case, a single adult contributed to 26 (11%) of the sampled offspring in the Hotel1 waterbody.

**FIGURE 4:**
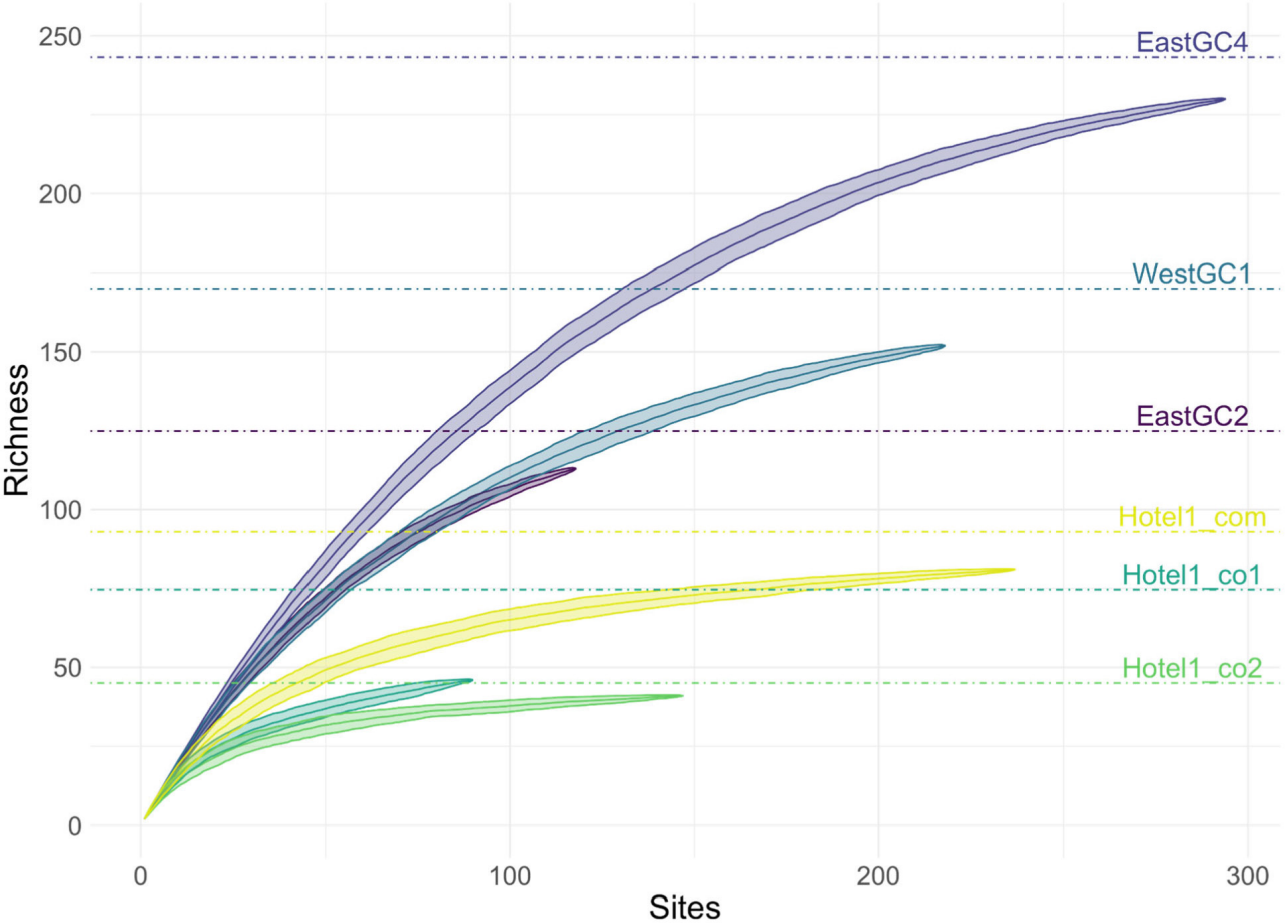
Parentage accumulation curves for the estimates of the asymptotic number of *P. clarkii* adults based on pedigree accumulation analysis (Sard et al. 2021) in four sites using the defined juvenile cohorts (East Golf Course 2, East Golf Course 4, West Golf Course 1, Hotel1 cohort one (co1), two (co2), and both cohorts combined (com)). Dashed horizontal lines are Chao estimates of the asymptotic number of adults, $\hat{N_{s}}$, see Table 1 for details. Estimates were based on 930 SNPs.

Multiple estimated measures of the number of breeding adults show consistent patterns across waterbodies. Estimates of the effective number of breeders, *N*_b_LD_ and *N*_b_Wang_, were both largest for EastGC4 (*N*_b_LD_ = 197.3 (jackknife confidence interval, CI = 154.7–261.8) and *N*_b_Wang_ = 253 (95% confidence interval, CI = 211–302)) and lowest for Hotel1 cohort 2, which again was collected after a pyrethrin‐based pesticide treatment (*N*_b_LD_ = 17.6 (CI = 15.3–20.3) and *N*_b_Wang_ = 24 (CI = 14–43); Table 1). While the two estimates differed in magnitude, they produced the same rank order across waterbodies from largest to smallest effective number of breeders. Overall, the Hotel1 cohorts had the lowest *N*_b_LD_ and *N*_b_Wang_, the EastGC ponds had the highest estimates, and the WestGC1 cohort was consistently intermediate (Table 1). The asymptotic number of successfully breeding adults ($\hat{N_{s}}$) largely showed the same pattern as *N*_b_LD_ and *N*_b_Wang_, except WestGC1 had the second largest $\hat{N_{s}}$, whereas EastGC2 had the largest *N*_b_ (Table 1, Figure 4). We did not find a significant relationship between our estimates of successfully reproducing adults and catch per unit effort in August 2020, or August 2021, for Hotel1 cohort 2 (Rho_Nb_ = 0.2, *P*_Nb_ = 0.78; Rho_Nb_wang_ = 0.2, *P*_Nb_wang_ = 0.78; Rho_chao_ = 0.4, *P*_chao_ = 0.75). Reproductive success of parents in the Hotel1 pond ranged from 1 to 26 offspring and was higher than that of parents from the two EastGC ponds, which ranged from 1 to 5 and 1 to 9 for EastGC2 and EastGC4, respectively. The ranking of the rarefied mean RS estimates showed the opposite pattern as *N*_b_ and $\hat{N_{s}}$. Confidence intervals for reproductive success did not overlap across waterbodies suggesting that they were significantly different, save for EastGC2 and WestGC1 and Hotel1 cohorts one and two.

We used inferred parentage to document whether parents were contributing to multiple cohorts. We found 10 parents with juvenile offspring in both Hotel1 cohorts (Table S3). All 53 juvenile pairs that included members of different age cohorts were categorized as half‐siblings (Table S3). Similarly, to document whether parents were contributing to offspring captured in more than one waterbody as a measure of gene flow, we compared inferred parents from multiple geographically proximal waterbodies, EastGC2 to EastGC4. We found 39 parents with juvenile offspring collected from both EastGC2 and EastGC4 (Table S4). Nearly all related juvenile pairs originating from a parent that reproduced in both waterbodies were categorized as half‐siblings (93 half‐sibling pairs). We identified two full‐sibling pairs (EastGC4‐J172‐22 and EastGC2‐J22, and EastGC4‐J172‐22 and EastGC2‐J46).

### 3.5 Evidence of Multiple Paternity

**FIGURE 5:**
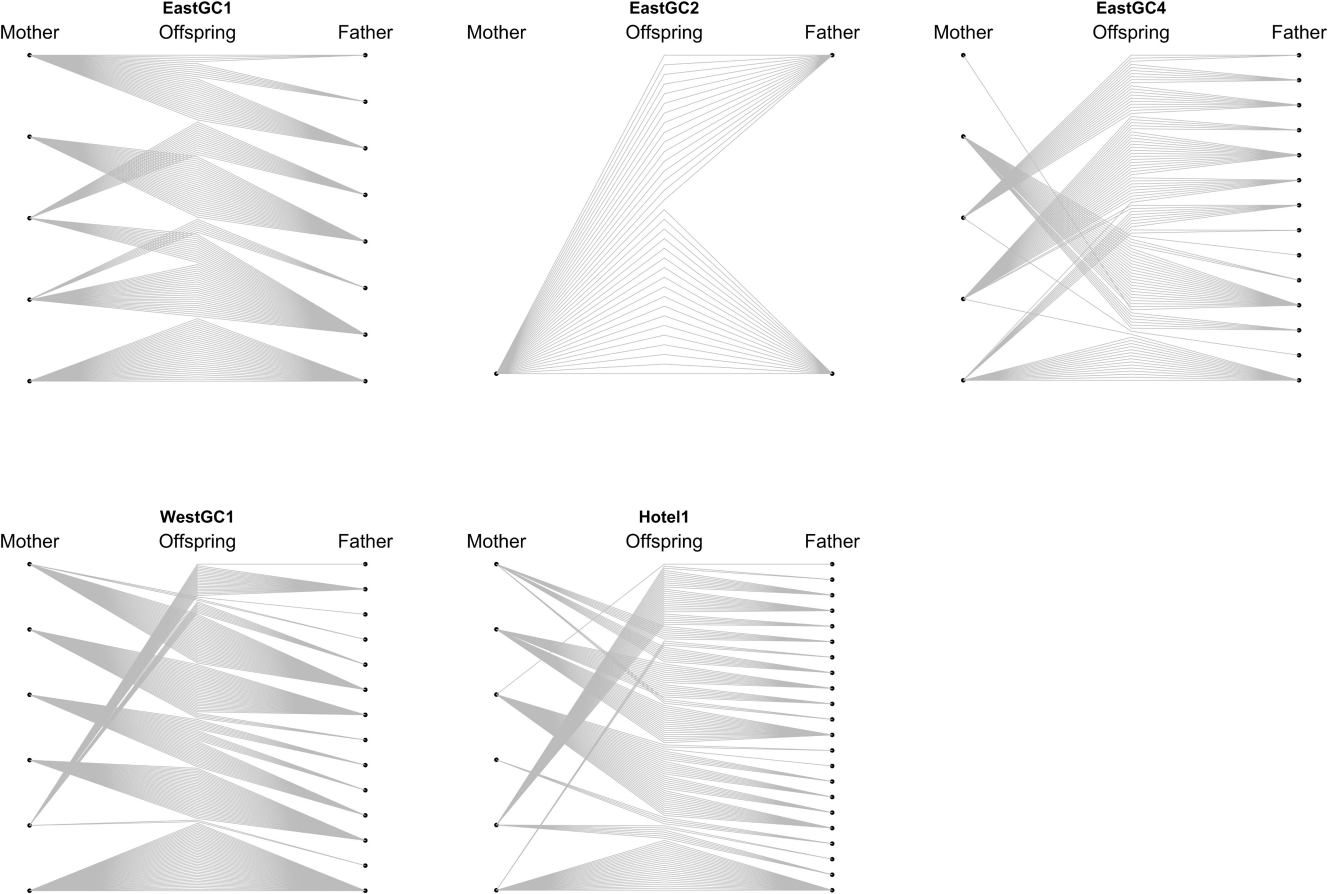
Visualization of reconstructed *Procambarus clarkii* pedigrees based on berried females and their offspring for five sites (East Golf Course 1, East Golf Course 2, East Golf Course 4, West Golf Course 1, and Hotel1). The center points represent genotyped hatchling and/or egg offspring. Lines indicate relationships among known genotyped mothers, offspring, and inferred mates. Multiple mates assigned to the offspring of an individual berried female is evidence for multiple paternity.

We documented instances of multiple paternity in all five waterbodies sampled (Table 2, Figure 5) based on an average of 30 ± 8 (mean ± SD) sampled offspring per berried female. The number of inferred mates ranged from 1 to 7 per berried female with an average of 2.8 ± 1.7 (mean ± SD) mates. Berried females from Hotel1 and EastGC4 had the highest average number of mates (3.67 ± 2.34 and 3.6 ± 1.5, respectively). Berried females from EastGC1 had the lowest average number of mates (1.8 ± 0.84). For example, the panel of the berried female from EastGC2 shows that its offspring were sired by two different inferred mates (Figure 5). One juvenile offspring from EastGC4 was assigned to an inferred female not included in our dataset, suggesting a genotyping and/or assignment error.

**TABLE 2:** Estimates of incidence of multiple paternity, including the number of identified inferred red swamp crayfish (*Procambarus clarkii*) males contributing to offspring for berried females from five sites (East Golf Course 1 (GC1), East Golf Course 1 (GC2), East Golf Course 4 (GC4), West Golf Course 1 (GC1), and Hotel1).

Site	Berried female	Years collected	No. screened offspring	No. inferred males
EastGC1	1	2021	30	1
	2	2021	31	2
	3	2021	29	1
	4	2021	31	2
	5	2021	31	3
Mean ± SD			30.4 ± 0.89	1.8 ± 0.84
EastGC2	1	2020	34	2
EastGC4	1	2020	26	4
	2	2020	29	5
	3	2020	21	4
	4	2021	31	4
Mean ± SD			26.8 ± 4.35	4.3 ± 0.50
WestGC1	1	2021	44	1
	2	2021	35	2
	3	2021	36	3
	4	2021	30	4
	5	2020	33	1
	6	2021	31	3
Mean ± SD			34.8 ± 5.04	2.33 ± 1.21
Hotel1	1	2020	28	2
	2	2021	18	3
	3	2020	36	6
	4	2020	4	1
	5	2020	36	7
	6	2020	36	3
Mean ± SD			26.0 ± 12.77	3.7 ± 2.34

*Note:* Below each waterbody with more than one berried female is the mean andSD.

We did not detect a relationship between body size (carapace length) of berried females and the number of inferred mates (Table S5). Based on the models evaluated, the best model (lowest AIC value) to describe differences in degree of multiple paternity included the number of offspring genotyped, the average August CPUE, and the year the berried female was collected (AIC = 74.87, Table S5). The number of inferred mates decreased from 2020 to 2021 (*p* = 0.01) and decreased with increasing August CPUE (*p* = 0.03). The number of offspring genotyped had a positive trend with the number of inferred mates but was not significant (*p* = 0.19). Isolating the relationship of just the number of mates and berried female body size showed no relationship but did suggest a potential difference between waterbodies (Figure S3). This may suggest that an interaction between female body size and waterbody could be an important term in future modeling of this relationship. Our sample sizes precluded us from having the power to use interaction terms in the analyses.

## 4 Discussion

### 4.1 Genomic Tools Provide Insights Into *P. clarkii* Reproductive Biology

The RAD capture genotyping panel we developed provided a means of characterizing *P. clarkii* reproductive biology, including aspects that require linking family groups across generations. We documented frequent multiple paternity within individual females' clutches, extending evidence of this behavior to the North American *P. clarkii* invaded range (Huner and Barr 1991; Yue et al. 2010). Multiple paternity may also provide an advantage allowing them to avoid genetic bottlenecks and inbreeding depression (Hosken and Stockley 2003; Holman and Kokko 2013), as has been documented for other invasive crayfishes (Kahrl, Laushman, and Roles 2014; Francesconi et al. 2021). The genotyping panel developed here allowed us to track parentage over multiple years, which can provide managers with estimates of the adult population contributing to recruitment across cohorts and seasons, including the timing of reproductive events. We used this capability to estimate spatial and temporal variability in the number of adult *P. clarkii* contributing to various cohorts (mean ± SD ranges from 45.1 ± 4.0 to 243.2 ± 5.5). Moreover, we quantified the effects of a pesticide treatment aimed at eradication in Hotel1, finding a nearly 40% (39.5%) decline in reproducing adults that appeared to be associated with a concomitant 45% increase in the average reproductive success of contributing adults. This suggests a density‐dependent compensatory reproductive response to the treatment, and that the treatment did not result in a net decline in recruitment potential despite the decline in adult abundance. The CPUE estimate for August 2021, after the treatment, was the lowest CPUE estimate (0.03) for Hotel1 from June to August over the years studied, supporting the decline in population size (number of reproducing adults). However, the increase in reproductive success highlights the recovery potential of the population and the need to conduct multiple treatments in order to increase the likelihood of eradication.

We demonstrate here that the kinship‐based method has the potential to further our understanding of age structure in Cambarid crayfishes. Aging methodologies in this group are notoriously unreliable due to repeated molting that leads to a lack of permanent age‐determining structures, such as scales or otoliths that are often used to age fish (France, Holmes, and Lynch 1991; Belchier et al. 1998; Vogt 2012). Current methods for determining age in crayfish are labor‐ and time‐intensive, such as longitudinal observations in captivity, capture–mark–recapture, or calibrated lipofuscin‐based aging (France, Holmes, and Lynch 1991; Belchier et al. 1998; Vogt 2012). Due to the inconsistency in aging crayfish, there is substantial uncertainty in the generation time of *P. clarkii* in invaded ecosystems. Generation time is an important indicator of population dynamics that can inform management efforts and basic organismal biology (González‐Calderón et al. 2023). In particular, generation time can inform the likelihood of successful crayfish population eradication. For example, populations of invasive North American beaver (*Castor canadensis*) in Argentina had lower generation times and started breeding at an earlier age where there was active invasive species management compared to areas that did not, which could lead to higher reproductive rates (González‐Calderón et al. 2023). Therefore, more accurate estimates of aging, growth, and generation time for *P. clarkii* could be derived from using the reconstructed pedigree method in juveniles paired with size–frequency distributions from routine monitoring. Further inference can be derived through longitudinal evaluation of contributing parents over multiple years. By combining genotyped juveniles over multiple years and inferring their pedigrees, we could estimate how many and how often adults reproduced over an extended period of time providing agencies with data on how well eradication efforts are working and if populations are adjusting to control efforts such as changing reproductive strategies or timing. This study adds to the growing body of literature that shows the utility of genetic tools in understanding reproductive ecology and movement of invasive species.

### 4.2 Multiple Juvenile Cohorts can Inform Treatment Timing

Our analysis of juvenile cohorts suggests that the majority of reproduction is synchronized in the late summer and early fall. The smallest juveniles were captured starting in August of both years, with larger individuals captured later in the fall and the following spring. Genetic data align with reports of reproductive timing of *P. clarkii* in Lake Trasimeno, Italy (Dörr et al. 2006), which lies at a similar latitude as the Michigan invasion, as well as Washington, USA (Mueller 2007). Additionally, the finding of full‐sibling pairs captured during fall and spring sampling suggests that juvenile crayfish captured in the spring were likely hatched the previous fall. Genetic data were consistent with observations in other invaded ranges, where a large reproductive bout corresponded with warmer temperatures (e.g., Spain, Gutiérrez‐Yurrita and Montes 1999). In their native range in warmer climates, *P. clarkii* may reproduce more consistently throughout the year when conditions are favorable (Huner and Barr 1991). Information about the timing of reproduction will help inform ongoing eradication efforts in Michigan. Whole‐pond pesticide treatments implemented prior to reproductive events (~June–July) or after juvenile emergence from burrows (~August–September) would likely result in the greatest effect. Further refinement of treatment strategies under conditions imposed by external factors, such as climate change, is prudent because they are likely to affect the timing of reproduction.

### 4.3 Intersite Connectivity Based on Juvenile Relatedness

We found related juvenile siblings in two different waterbodies (EastGC2 and EastGC4), indicating that there is some movement of adults or juveniles between geographically proximal (~128 m apart) waterbodies. Most related pairs were identified as half‐siblings and were collected in the fall of 2020 and the spring of 2021; thus, they were in the same cohort for each waterbody. In 21 of the 25 sibling groups that had juveniles collected in both the fall of 2020 and the spring of 2021, individuals collected in the spring had larger carapace lengths. We also found evidence for two full‐sibling pairs across the sampled waterbodies (Table S5, Figure 1). Two EastGC2 juveniles collected in the fall of 2020 (carapace lengths 13 mm and 18 mm) were inferred to be the full siblings of the same juvenile collected in the spring of 2021 in EastGC4 (carapace length 19 mm). This pattern could be due to berried females moving between locations, males moving between locations, sperm storage, or the dispersal of juvenile crayfish. Overland dispersal often occurs during peak breeding, including dispersal of berried females that move overland (Ramalho and Anastácio 2015). Generally, *P. clarkii* can disperse tens of meters over land per day (Gherardi, Barbaresi, and Salvi 2000; Gherardi, Tricarico, and Ilhéu 2002; Barbaresi et al. 2004; Aquiloni, Ilhéu, and Gherardi 2005). It is also possible that our pedigree reconstructions falsely inferred sibling relationships for pairs of individuals. Previous studies illustrate challenges with inferences based on half‐sibling relationships, particularly in SNP‐based pedigree reconstructions (e.g., Ackerman et al. 2017; Baetscher et al. 2018; Sard et al. 2020). Nonetheless, full‐sibling inferences are expected to be highly accurate when based on large SNP datasets, as used in this study. Thus, our observations of full‐sibling pairs across waterbodies provide strong evidence of dispersal on a local scale. With additional future sampling, multigenerational pedigree reconstructions and “recaptures” of close kin can further reveal details concerning gene flow among the waterbodies and the importance of intervening landscape features (Prystupa et al. 2021; Delaval et al. 2023; Schmidt et al. 2023).

### 4.4 Multiple Paternity in *P. clarkii*

Data revealed that the majority (77%) of the berried females mated with multiple males according to offspring pedigrees. Our overall rate of multiple paternity is below the 97% found in populations in China, and our rates of inferred mates per female (1–7) are broader than previous estimates (2–4; Yue et al. 2010). In our study area, one male often sired the majority of the offspring while additional males were assigned to fewer offspring of the same clutch, which supports previous work indicating parentage of dominant males (Yue et al. 2010). Our results support a growing body of literature that suggests multiple paternity is advantageous in invasive species that are successfully established, such as in the American mosquitofish *Gambusia holbrooki* (Zeng et al. 2017), marine gastropods *Crepidula fornicata* (Le Cam et al. 2009) and *Littorina saxatilis* (Rafajlović et al. 2013), two rat species (*Rattus norvegicus* and *R. rattus*; Miller et al. 2010), and in the spotted lanternfly *Lycorma delicatula* (Belouard and Behm 2023). Despite often coming at a cost (time, energy, and risk of injury) to females (Slatyer et al. 2012), multiple matings can provide benefits, including increased rates of heterozygosity (Holman and Kokko 2013; Rafajlović et al. 2013; Taylor, Price, and Wedell 2014) and lower interindividual relatedness within cohorts (Chesser 1991a, 1991b). This effect of multiple matings on heterozygosity can be especially substantial in small, isolated populations (Rafajlović et al. 2013), which can help overcome the loss of genetic diversity often caused by founder bottlenecks in colonizing populations. It should also be noted that a potential incident of parthenogenesis— the development of embryos from unfertilized eggs—was recorded in *P. clarkii*, but the results were based on only five microsatellites (Yue et al. 2008). Furthermore, an invasive congener species, *Procambarus virginalis*, is known to undergo parthenogenesis (Scholtz et al. 2002; Chucholl, Morawetz, and Groß 2012), as well as another Cambarid species, *Faxonius limosus* (synonym *Orconectes limosus*) (Buřič et al. 2011), providing support that it may also be a strategy used in *P. clarkii*. Cloning is a beneficial strategy for successful non‐native range invasions as shown in the crayfish *P. virginalis* (Gutekunst et al. 2018), the freshwater snail *Potamopyrgus antipodarum* (Verhaegen, von Jungmeister, and Haase 2021), and multiple Hymenoptera species (Queffelec et al. 2021).

We did not find that multiple paternity was correlated with adult female body size (carapace length), a measure of female reproductive “quality.” Our low sample size of berried females and using only a subset of offspring, rather than all available offspring, may hinder our power to make strong conclusions. However, our result is consistent with previous work that found the number of inferred mates was unrelated to female total body length (Yue et al. 2010). *Procambarus clarkii* has been shown to have both male and female mate choice (Aquiloni and Gherardi 2008a, 2008b, 2008c; Hamasaki, Nishimoto, and Dan 2022), potentially including sperm competition (McLay and van den Brink 2016), which could contribute to this result.

### 4.5 Monitoring Effective Population Sizes for Management

Estimates of effective population size (*N*_e_), the effective number of breeding adults (*N*_b_), or the minimum number of spawning adults (*N*_s_) can provide useful information in conservation and management contexts (Schwartz, Luikart, and Waples 2006; Osborne et al. 2010; Tallmon et al. 2010; Luikart et al. 2021; Weise et al. 2022). In this study, we found evidence for moderate declines in *N*_b_ and *N*_s_ following extensive control and removal actions targeting *P. clarkii* at the Hotel1 site (Table 1). However, we found no significant correlation between genetic estimates of abundance and CPUE from trapping efforts across a limited number of sampled waterbodies. Estimates of per‐individual reproductive success increased in Hotel1 cohort 2, potentially indicating the effects of density‐dependent processes in established populations and obscuring expected relationships between *N*_b_ and census size. Even in the absence of clear connections to census population sizes, estimates of parameters like *N*_b_ and *N*_s_ can provide important insights on invasive species reproductive biology, ecology, and control (e.g., Bingham, Sepulveda, and Painter 2021; Taylor, Bangs, and Long 2021; Weise et al. 2023). Our results not only highlight the potential utility of genetic data for monitoring species invasions but also illustrate associated challenges and limitations, such as our modest number of populations to detect a potential relationship between abundance estimates and CPUE.

### 4.6 Future Directions

The RAD capture genotyping panel we developed provided a means of characterizing *P. clarkii* reproductive biology, including aspects that require linking family groups across generations. For example, we document high levels of multiple paternity, which may allow RSC to avoid genetic bottlenecks and loss of genetic diversity. These findings are important because they suggest that deleterious effects of inbreeding (depression) that are more likely with single pair matings when population size is small will be unlikely during founding events and expansion into new habitats following colonization.

Managers would benefit from methods that allow quantitative assessment of the effects of control actions. Here, we quantified the effects of a pesticide treatment aimed at eradication based on pedigree analyses of offspring before and following treatment. We documented a decline in the number of reproducing adults following control efforts in Hotel1. However, data also revealed an apparent increase in the average reproductive success of remaining and successfully reproducing adults, suggesting a possible density‐dependent compensatory reproductive response to the treatment. The interpretation of the findings is that the treatment did not result in a net decline in recruitment potential despite declines in adult abundance. Generally, we found evidence for a high reproductive skew in the waterbodies sampled, demonstrating high variation in male reproductive success inferred from pedigrees of offspring from berried females. Further analyses, for example, parentage analyses that focus on aspects of male phenotypes such as body size that explain the observed skew in male reproductive analysis, would be useful.

The SNP panel developed for this study and pedigree analyses performed allowed estimation of the effective breeding number of adults in each waterbody surveyed as well as estimates of the number of breeding adults consistent with offspring produced. These estimators are useful surrogates to estimates of census population size, which is a critical parameter to estimate *P. clarkii* levels of recruitment (population growth potential) and response to control measures (population decline and likelihood of extirpation). Moving forward, the recent developments in parentage‐based tagging (Steele et al. 2019) and adult–juvenile close‐kin analyses (Bravington, Skaug, and Anderson 2016) provide unparalleled opportunities to characterize *P. clarkii* population dynamics and ecology. These “next generation” methods expand upon widely used capture–mark–recapture methods based on genetic measures of identity (e.g., American marten, *Martes americana*; Williams et al. 2009); American black bear, *Ursus americanus* (Dreher et al. 2007). Importantly, genetic estimators do not rely on the release and recapture of individuals for census number estimation, which is important for invasive species management. We also demonstrate here that the kinship‐based method has the potential to further our understanding of age structure in Cambarid crayfishes. For example, our analysis of juvenile cohorts suggests the majority of reproduction is synchronized in the late summer and early fall.

Autocidal techniques are seen as a potential solution to reduce reproductive output that can help control invasive populations and have been proposed for invasive *P. clarkii* populations. However, X‐ray irradiation to sterilize males has limited effectiveness and feasibility (Aquiloni et al. 2009; Manfrin et al. 2021), and the technology to produce neo‐females, such as those for invasive prawns (Rungsin, Paankhao, and Na‐Nakorn 2006; Parana et al. 2022), has yet to be realized for *P. clarkii*. The effectiveness of autocidal techniques in invasive *P. clarkii* may be affected by their mating system. Mate choice based on size may inform which males and females would be best to sterilize (Hamasaki, Tsuboi, and Dan 2022). Generally, polyandry may make sterilized males a less effective control strategy than if *P. clarkii* females mated with only one male, depending on the mechanism of sterilization.

## Conflicts of Interest

The authors declare no conflicts of interest.

## Supporting information


Appendix S1.

## Data Availability

Individual sequences after demultiplexing libraries are available on the NCBI Sequence Read Archive under Bioproject PRJNA1148680. Code used for bioinformatics and data analysis is available at https://github.com/NicoleAdams‐sci/RedSwampCrayfish_reproBio.

## References

[eva70007-bib-0001] Ackerman, M. S. , P. Johri , K. Spitze , et al. 2017. “Estimating Seven Coefficients of Pairwise Relatedness Using Population‐Genomic Data.” Genetics 206: 105–118. 28341647 10.1534/genetics.116.190660 PMC5419463

[eva70007-bib-0002] Alcorlo, P. , W. Geiger , and M. Otero . 2008. “Reproductive Biology and Life Cycle of the Invasive Crayfish *Procambarus clarkii* (Crustacea: Decapoda) in Diverse Aquatic Habitats of South‐Western Spain: Implications for Population Control.” Fundamental and Applied Limnology 173: 197–212.

[eva70007-bib-0003] Ali, O. A. , S. M. O'Rourke , S. J. Amish , et al. 2016. “RAD Capture (Rapture): Flexible and Efficient Sequence‐Based Genotyping.” Genetics 202: 389–400. 26715661 10.1534/genetics.115.183665 PMC4788223

[eva70007-bib-0004] Anderson, J. H. , P. L. Faulds , K. D. Burton , M. E. Koehler , W. I. Atlas , and T. P. Quinn . 2015. “Dispersal and Productivity of Chinook (*Oncorhynchus Tshawytscha*) and Coho (*Oncorhynchus Kisutch*) Salmon Colonizing Newly Accessible Habitat.” Canadian Journal of Fisheries and Aquatic Sciences 72: 454–465.

[eva70007-bib-0005] Andrews, K. R. , P. A. Hohenlohe , M. R. Miller , B. K. Hand , J. E. Seeb , and G. Luikart . 2014. “Trade‐Offs and Utility of Alternative RADseq Methods: Reply to Puritz et al.” Molecular Ecology 23: 5943–5946. 25319129 10.1111/mec.12964

[eva70007-bib-0006] Aquiloni, L. , A. Becciolini , R. Berti , S. Porciani , C. Trunfio , and F. Gherardi . 2009. “Managing Invasive Crayfish: Use of X‐Ray Sterilisation of Males.” Freshwater Biology 54: 1510–1519.

[eva70007-bib-0007] Aquiloni, L. , and F. Gherardi . 2008a. “Assessing Mate Size in the Red Swamp Crayfish *Procambarus clarkii*: Effects of Visual Versus Chemical Stimuli.” Freshwater Biology 53: 461–469.

[eva70007-bib-0008] Aquiloni, L. , and F. Gherardi . 2008b. “Evidence of Female Cryptic Choice in Crayfish.” Biology Letters 4: 163–165. 18270163 10.1098/rsbl.2007.0590 PMC2429928

[eva70007-bib-0009] Aquiloni, L. , and F. Gherardi . 2008c. “Mutual Mate Choice in Crayfish: Large Body Size Is Selected by Both Sexes, Virginity by Males Only.” Journal of Zoology 274: 171–179.

[eva70007-bib-0010] Aquiloni, L. , M. Ilhéu , and F. Gherardi . 2005. “Habitat Use and Dispersal of the Invasive Crayfish *Procambarus clarkii* in Ephemeral Water Bodies of Portugal.” Marine and Freshwater Behaviour and Physiology 38: 225–236.

[eva70007-bib-0011] Athrey, G. , T. K. Hodges , M. R. Reddy , H. J. Overgaard , and A. Matias . 2012. “The Effective Population Size of Malaria Mosquitoes: Large Impact of Vector Control.” PLoS Genetics 8: 1003097. 10.1371/journal.pgen.1003097 PMC3521722 23271973

[eva70007-bib-0012] Baetscher, D. S. , E. C. Anderson , E. A. Gilbert‐Horvath , et al. 2019. “Dispersal of a Nearshore Marine Fish Connects Marine Reserves and Adjacent Fished Areas Along an Open Coast.” Molecular Ecology 28: 1611–1623. 30739378 10.1111/mec.15044

[eva70007-bib-0013] Baetscher, D. S. , A. J. Clemento , T. C. Ng , E. C. Anderson , and J. C. Garza . 2018. “Microhaplotypes Provide Increased Power From Short‐Read DNA Sequences for Relationship Inference.” Molecular Ecology Resources 18: 296–305. 29143457 10.1111/1755-0998.12737

[eva70007-bib-0014] Barbaresi, S. , G. Santini , E. Tricarico , and F. Gherardi . 2004. “Ranging Behaviour of the Invasive Crayfish, *Procambarus clarkii* (Girard).” Journal of Natural History 38: 2821–2832.

[eva70007-bib-0015] Bazin, É. , H. Mathé‐Hubert , B. Facon , J. Carlier , and V. Ravigné . 2014. “The Effect of Mating System on Invasiveness: Some Genetic Load May Be Advantageous When Invading New Environments.” Biological Invasions 16: 875–886.

[eva70007-bib-0016] Belchier, M. , L. Edsman , M. R. J. Sheehy , and P. M. J. Shelton . 1998. “Estimating Age and Growth in Long‐Lived Temperate Freshwater Crayfish Using Lipofuscin.” Freshwater Biology 39: 439–446.

[eva70007-bib-0017] Belouard, N. , and J. E. Behm . 2023. “Multiple Paternity in the Invasive Spotted Lanternfly (Hemiptera: Fulgoridae).” Environmental Entomology 52: 949–955. 37611175 10.1093/ee/nvad083

[eva70007-bib-0018] Bingham, D. M. , A. J. Sepulveda , and S. Painter . 2021. “A Small Proportion of Breeders Drive American Bullfrog Invasion of the Yellowstone River Floodplain, Montana.” Northwest Science 94: 231–242.

[eva70007-bib-0019] Bolger, A. M. , M. Lohse , and B. Usadel . 2014. “Trimmomatic: A Flexible Trimmer for Illumina Sequence Data.” Bioinformatics 30: 2114–2120. 24695404 10.1093/bioinformatics/btu170 PMC4103590

[eva70007-bib-0020] Bravington, M. V. , P. M. Grewe , and C. R. Davies . 2016. “Absolute Abundance of Southern Bluefin Tuna Estimated by Close‐Kin Mark‐Recapture.” Nature Communications 17: 1–8. 10.1038/ncomms13162 PMC5114523 27841264

[eva70007-bib-0021] Bravington, M. V. , H. J. Skaug , and E. C. Anderson . 2016. “Close‐Kin Mark‐Recapture.” Statistical Science 31: 259–274.

[eva70007-bib-0022] Bretman, A. , D. Newcombe , and T. Tregenza . 2009. “Promiscuous Females Avoid Inbreeding by Controlling Sperm Storage.” Molecular Ecology 18: 3340–3345. 19694961 10.1111/j.1365-294X.2009.04301.x

[eva70007-bib-0023] Budnick, W. R. , D. Hayes , S. Herbst , et al. 2024. “Factors Influencing Detection of Invasive Red Swamp Crayfish *Procambarus clarkii* (Girard, 1852) in Michigan Ponds.” Hydrobiologia 851: 2761–2774.

[eva70007-bib-0024] Budnick, W. R. , B. Roth , L. R. Nathan , et al. 2022. “Evaluation of Five Trap Designs for Removal of Invasive Red Swamp Crayfish (*Procambarus clarkii* Girard, 1852) in Southern Michigan: Catch per Unit Effort, Body Size, and Sex Biases.” Management of Biological Invasions 13: 369–390.

[eva70007-bib-0025] Buřič, M. , M. Hulák , A. Kouba , A. Petrusek , and P. Kozák . 2011. “A Successful Crayfish Invader Is Capable of Facultative Parthenogenesis: A Novel Reproductive Mode in Decapod Crustaceans.” PLoS One 6: e20281. 21655282 10.1371/journal.pone.0020281 PMC3105005

[eva70007-bib-0026] Catchen, J. , P. A. Hohenlohe , S. Bassham , A. Amores , and W. A. Cresko . 2013. “Stacks: An Analysis Tool set for Population Genomics.” Molecular Ecology 22: 3124–3140. 23701397 10.1111/mec.12354 PMC3936987

[eva70007-bib-0027] Cecchinelli, E. , L. Aquiloni , G. Maltagliati , G. Orioli , E. Tricarico , and F. Gherardi . 2012. “Use of Natural Pyrethrum to Control the Red Swamp Crayfish *Procambarus clarkii* in a Rural District of Italy.” Pest Management Science 68: 839–844. 22396306 10.1002/ps.2335

[eva70007-bib-0028] Chesser, R. K. 1991a. “Gene Diversity and Female Philopatry.” Genetics 127: 437–447. 2004714 10.1093/genetics/127.2.437 PMC1204371

[eva70007-bib-0029] Chesser, R. K. 1991b. “Influence of Gene Flow and Breeding Tactics on Gene Diversity Within Populations.” Genetics 129: 573–583. 1743493 10.1093/genetics/129.2.573 PMC1204645

[eva70007-bib-0030] Chucholl, C. 2011. “Population Ecology of an Alien “Warm Water” Crayfish (*Procambarus clarkii*) in a New Cold Habitat.” Knowledge and Management of Aquatic Ecosystems 401: 29.

[eva70007-bib-0031] Chucholl, C. 2013. “Invaders for Sale: Trade and Determinants of Introduction of Ornamental Freshwater Crayfish.” Biological Invasions 15: 125–141.

[eva70007-bib-0032] Chucholl, C. , K. Morawetz , and H. Groß . 2012. “The Clones Are Coming—Strong Increase in Marmorkrebs [*Procambarus Fallax* (Hagen, 1870) f. *Virginalis*] Records From Europe.” Aquatic Invasions 7: 511–519.

[eva70007-bib-0033] Dale, R. K. , B. S. Pedersen , and A. R. Quinlan . 2011. “Pybedtools: A Flexible Python Library for Manipulating Genomic Datasets and Annotations.” Bioinformatics 27: 3423–3424. 21949271 10.1093/bioinformatics/btr539 PMC3232365

[eva70007-bib-0034] Danecek, P. , A. Auton , G. Abecasis , et al. 2011. “The Variant Call Format and VCFtools.” Bioinformatics 27: 2156–2158. 21653522 10.1093/bioinformatics/btr330 PMC3137218

[eva70007-bib-0035] Delaval, A. , V. Bendall , S. J. Hetherington , et al. 2023. “Evaluating the Suitability of Close‐Kin Mark‐Recapture as a Demographic Modelling Tool for a Critically Endangered Elasmobranch Population.” Evolutionary Applications 16: 461–473. 36793682 10.1111/eva.13474 PMC9923483

[eva70007-bib-0036] Do, C. , R. S. Waples , D. Peel , G. M. Macbeth , B. J. Tillett , and J. R. Ovenden . 2014. “NeEstimator v2: Re‐Implementation of Software for the Estimation of Contemporary Effective Population Size (*N* _ *e* _) From Genetic Data.” Molecular Ecology Resources 14: 209–214. 23992227 10.1111/1755-0998.12157

[eva70007-bib-0037] Dörr, A. J. M. , G. La Porta , G. Pedicillo , and M. Lorenzoni . 2006. “Biology of *Procambarus clarkii* (Girard, 1852) in Lake Trasimeno.” Bulletin français de la pêche et de la pisciculture 380: 1155–1167.

[eva70007-bib-0038] Dreher, B. P. , S. R. Winsterstein , K. T. Scribner , et al. 2007. “Noninvasive Estimation of Black Bear Abundance Incorporating Genotyping Errors and Harvested Bear.” Journal of Wildlife Management 71: 2684–2693.

[eva70007-bib-0039] Evangelista, C. , R. J. Britton , and J. Cucherousset . 2015. “Impacts of Invasive Fish Removal Through Angling on Population Characteristics and Juvenile Growth Rate.” Ecology and Evolution 5: 2193–2202. 26078856 10.1002/ece3.1471 PMC4461421

[eva70007-bib-0040] Evans, M. L. , M. A. Johnson , D. Jacobson , J. Wang , M. Hogansen , and K. G. O'malley . 2016. “Evaluating a Multi‐Generational Reintroduction Program for Threatened Salmon Using Genetic Parentage Analysis.” Canadian Journal of Fisheries and Aquatic Sciences 73: 844–852.

[eva70007-bib-0041] Fantle‐Lepczyk, J. E. , P. J. Haubrock , A. M. Kramer , et al. 2022. “Economic Costs of Biological Invasions in the United States.” Science of the Total Environment 806: 151318. 34743879 10.1016/j.scitotenv.2021.151318

[eva70007-bib-0042] Ford, M. J. , A. Murdoch , and M. Hughes . 2015. “Using Parentage Analysis to Estimate Rates of Straying and Homing in Chinook Salmon (*Oncorhynchus Tshawytscha*).” Molecular Ecology 24: 1109–1121. 25626589 10.1111/mec.13091

[eva70007-bib-0043] France, R. , J. Holmes , and A. Lynch . 1991. “Use of Size–Frequency Data to Estimate the Age Composition of Crayfish Populations.” Canadian Journal of Fisheries and Aquatic Sciences 48: 2324–2332.

[eva70007-bib-0044] Francesconi, C. , M. Pîrvu , A. Schrimpf , R. Schulz , L. Părvulescu , and K. Theissinger . 2021. “Mating Strategies of Invasive Versus Indigenous Crayfish: Multiple Paternity as a Driver for Invasion Success?” Freshwater Crayfish 26: 89–98.

[eva70007-bib-0045] Gallardo, B. , M. Clavero , M. I. Sánchez , and M. Vilà . 2016. “Global Ecological Impacts of Invasive Species in Aquatic Ecosystems.” Global Change Biology 22: 151–163. 26212892 10.1111/gcb.13004

[eva70007-bib-0046] Gherardi, F. , and P. Acquistapace . 2007. “Invasive Crayfish in Europe: The Impact of *Procambarus clarkii* on the Littoral Community of a Mediterranean Lake.” Freshwater Biology 52: 1249–1259.

[eva70007-bib-0047] Gherardi, F. , L. Aquiloni , J. Diéguez‐Uribeondo , and E. Tricarico . 2011. “Managing Invasive Crayfish: Is There a Hope?” Aquatic Sciences 73: 185–200.

[eva70007-bib-0048] Gherardi, F. , S. Barbaresi , and G. Salvi . 2000. “Spatial and Temporal Patterns in the Movement of *Procambarus clarkii*, an Invasive Crayfish.” Aquatic Sciences 20: 179–193.

[eva70007-bib-0049] Gherardi, F. , E. Tricarico , and M. Ilhéu . 2002. “Movement Patterns of an Invasive Crayfish, *Procambarus clarkii*, in a Temporary Stream of Southern Portugal.” Ethology Ecology and Evolution 14: 183–197.

[eva70007-bib-0050] Gibson, M. J. S. , D. J. Crawford , M. T. Holder , et al. 2020. “Genome‐Wide Genotyping Estimates Mating System Parameters and Paternity in the Island Species *Tolpis Succulenta* .” American Journal of Botany 107: 1189–1197. 32864742 10.1002/ajb2.1515

[eva70007-bib-0051] González‐Calderón, A. , J. Escobar , G. Deferrari , and A. Schiavini . 2023. “Demographic Plasticity in an Invasive Species: The Effects of Time Since Invasion and Population Management History on Beavers in Tierra del Fuego, Argentina.” Journal of Zoology 319: 175–187.

[eva70007-bib-0052] Green, S. J. , and E. D. Grosholz . 2021. “Functional Eradication as a Framework for Invasive Species Control.” Frontiers in Ecology and the Environment 19: 98–107.

[eva70007-bib-0053] Gutekunst, J. , R. Andriantsoa , C. Falckenhayn , et al. 2018. “Clonal Genome Evolution and Rapid Invasive Spread of the Marbled Crayfish.” Nature Ecology & Evolution 2: 567–573. 29403072 10.1038/s41559-018-0467-9

[eva70007-bib-0054] Gutiérrez‐Yurrita, P. J. , and C. Montes . 1999. “Bioenergetics and Phenology of Reproduction of the Introduced Red Swamp Crayfish, *Procambarus clarkii*, in Donana National Park, Spain, and Implications for Species Management.” Freshwater Biology 42: 561–574.

[eva70007-bib-0055] Hamasaki, K. , S. Nishimoto , and S. Dan . 2022. “Multiple Spawning of the Red Swamp Crayfish *Procambarus clarkii* (Decapoda: Cambaridae) Under Laboratory Conditions.” Invertebrate Reproduction and Development 2: 1–10.

[eva70007-bib-0056] Hamasaki, K. , T. Tsuboi , and S. Dan . 2022. “Effects of Body Size on Mating Behaviour and Spawning of the Red Swamp Crayfish *Procambarus clarkii* .” Aquatic Animals 19: AA2022.

[eva70007-bib-0057] Haubrock, P. J. , A. J. Turbelin , R. N. Cuthbert , et al. 2021. “Economic Costs of Invasive Alien Species Across Europe.” NeoBiota 67: 153–190.

[eva70007-bib-0058] Hobbs, H. H. , J. P. Jass , and J. V. Huner . 1989. “A Review of Global Crayfish Introductions With Particular Emphasis on Two North American Species (Decapoda, Cambaridae).” Crustaceana 56: 299–316.

[eva70007-bib-0059] Hohenlohe, P. A. , M. D. Day , S. J. Amish , et al. 2013. “Genomic Patterns of Introgression in Rainbow and Westslope Cutthroat Trout Illuminated by Overlapping Paired‐End RAD Sequencing.” Molecular Ecology 22: 3002–3013. 23432212 10.1111/mec.12239 PMC3664261

[eva70007-bib-0060] Holman, L. , and H. Kokko . 2013. “The Consequences of Polyandry for Population Viability, Extinction Risk and Conservation.” Philosophical Transactions of the Royal Society, B: Biological Sciences 368: 20120053. 10.1098/rstb.2012.0053 PMC3576587 23339244

[eva70007-bib-0061] Hosken, D. J. , and P. Stockley . 2003. “Benefits of Polyandry: A Life History Perspective.” In Evolutionary Biology, edited by R. J. MacIntyre and M. T. Clegg , 173–194. Boston, MA: Springer.

[eva70007-bib-0062] Huner, J. V. , and J. E. Barr . 1991. Red Swamp Crawfish: Biology and Exploitation. Third ed. Baton Rouge: NOAA.

[eva70007-bib-0063] Jones, O. R. , and J. Wang . 2010. “COLONY: A Program for Parentage and Sibship Inference From Multilocus Genotype Data.” Molecular Ecology Resources 10: 551–555. 21565056 10.1111/j.1755-0998.2009.02787.x

[eva70007-bib-0064] Kahrl, A. F. , R. H. Laushman , and A. J. Roles . 2014. “Evidence for Multiple Paternity in Two Species of *Orconectes* Crayfish.” Canadian Journal of Zoology 92: 985–988.

[eva70007-bib-0065] Klassen, W. , and C. F. Curtis . 2005. “History of the Sterile Insect Technique.” In Sterile Insect Technique: Principles and Practice in Area‐Wide Integrated Pest Management, 3–36. Dordrecht: Springer.

[eva70007-bib-0066] Le Cam, S. , J. A. Pechenik , M. Cagnon , and F. Viard . 2009. “Fast Versus Slow Larval Growth in an Invasive Marine Mollusc: Does Paternity Matter?” Journal of Heredity 100: 455–464. 19307296 10.1093/jhered/esp007

[eva70007-bib-0067] Li, H. 2013. “Aligning Sequence Reads, Clone Sequences and Assembly Contigs With BWA‐MEM.” *ArXiv* 1303.3997 [q‐Bio.GN].

[eva70007-bib-0068] Li, H. , and R. Durbin . 2010. “Fast and Accurate Long‐Read Alignment With Burrows‐Wheeler Transform.” Bioinformatics 26: 589–595. 20080505 10.1093/bioinformatics/btp698 PMC2828108

[eva70007-bib-0069] Li, H. , B. Handsaker , A. Wysoker , et al. 2009. “The Sequence Alignment/Map Format and SAMtools.” Bioinformatics 25: 2078–2079. 19505943 10.1093/bioinformatics/btp352 PMC2723002

[eva70007-bib-0070] Lidova, J. , M. Buric , A. Kouba , and J. Velisek . 2019. “Acute Toxicity of Two Pyrethroid Insecticides for Five Non‐indigenous Crayfish Species in Europe.” Veterinární Medicína 64: 125–133.

[eva70007-bib-0071] Lischer, H. E. L. , and L. Excoffier . 2012. “PGDSpider: An Automated Data Conversion Tool for Connecting Population Genetics and Genomics Programs.” Bioinformatics 28: 298–299. 22110245 10.1093/bioinformatics/btr642

[eva70007-bib-0072] Loureiro, T. G. , S. G. Anastácio , C. Souty‐Grosset , P. B. Araujo , and M. P. Almerão . 2015. “Red Swamp Crayfish: Biology, Ecology and Invasion—An Overview.” Nauplius 23: 1–19.

[eva70007-bib-0073] Luikart, G. , T. Antao , B. K. Hand , et al. 2021. “Detecting Population Declines via Monitoring the Effective Number of Breeders (*N* _b_).” Molecular Ecology Resources 21: 379–393. 32881365 10.1111/1755-0998.13251

[eva70007-bib-0074] Manfrin, C. , A. Giglio , L. Pallavicini , et al. 2021. “Medium‐Term Feasibility of the Management of the Invasive Crayfish *Procambarus clarkii* With the Sterile Males Release Technique.” Pest Management Science 77: 2494–2501. 33442899 10.1002/ps.6280

[eva70007-bib-0075] Manichaikul, A. , J. C. Mychaleckyj , S. S. Rich , K. Daly , M. Sale , and W. M. Chen . 2010. “Robust Relationship Inference in Genome‐Wide Association Studies.” Bioinformatics 26: 2867–2873. 20926424 10.1093/bioinformatics/btq559 PMC3025716

[eva70007-bib-0076] Marcy‐Quay, B. , S. A. Sethi , N. O. Therkildsen , and C. E. Kraft . 2020. “Expanding the Feasibility of Fish and Wildlife Assessments With Close‐Kin Mark–Recapture.” Ecosphere 11: e03259.

[eva70007-bib-0077] Martín‐Torrijos, L. , T. Kawai , J. Makkonen , J. Jussila , H. Kokko , and J. Diéguez‐Uribeondo . 2018. “Crayfish Plague in Japan: A Real Threat to the Endemic *Cambaroides Japonicus* .” PLoS One 13: e0195353. 29617418 10.1371/journal.pone.0195353 PMC5884544

[eva70007-bib-0078] Matsuzaki, S. I. S. , N. Usio , N. Takamura , and I. Washitani . 2009. “Contrasting Impacts of Invasive Engineers on Freshwater Ecosystems: An Experiment and Meta‐Analysis.” Oecologia 158: 673–686. 18941787 10.1007/s00442-008-1180-1

[eva70007-bib-0079] McKinney, G. J. , R. K. Waples , L. W. Seeb , and J. E. Seeb . 2017. “Paralogs Are Revealed by Proportion of Heterozygotes and Deviations in Read Ratios in Genotyping‐By‐Sequencing Data From Natural Populations.” Molecular Ecology Resources 17: 656–669. 27762098 10.1111/1755-0998.12613

[eva70007-bib-0080] McLay, C. L. , and A. M. van den Brink . 2016. “Crayfish Growth and Reproduction.” In Biology and Ecology of Crayfish, edited by M. Longshaw and P. Stebbing , 62–116. Boca Raton, MA: CRC Press.

[eva70007-bib-0081] Miller, S. D. , J. C. Russell , H. E. Macinnes , J. Abdelkrim , and R. M. Fewster . 2010. “Multiple Paternity in Wild Populations of Invasive *Rattus* Species.” New Zealand Journal of Ecology 34: 360–363.

[eva70007-bib-0082] Mrugała, A. , T. Kawai , E. Kozubíková‐Balcarová , and A. Petrusek . 2017. “ *Aphanomyces Astaci* Presence in Japan: A Threat to the Endemic and Endangered Crayfish Species *Cambaroides Japonicus*?” Aquatic Conservation 27: 103–114.

[eva70007-bib-0083] Mueller, K. W. 2007. “Reproductive Habits of Non‐native Red Swamp Crayfish (*Procambarus clarkii*) at Pine Lake, Sammamish, Washington.” Northwest Science 81: 246–250.

[eva70007-bib-0084] National Park Service . 2022. Invasive Aquatics. Washington, DC: National Park Service. https://www.nps.gov/subjects/invasive/aquatic‐invasive‐species.htm.

[eva70007-bib-0085] Oficialdegui, F. J. , M. I. Sánchez , and M. Clavero . 2020. “One Century Away From Home: How the Red Swamp Crayfish Took Over the World.” Reviews in Fish Biology and Fisheries 30: 121–135.

[eva70007-bib-0086] Oksanen, R. J. , G. L. Simpson , F. G. Blanchet , et al. 2022. “Vegan: Community Ecology Package. R package version 2.6‐2.” https://CRAN.R‐project.org/package=vegan.

[eva70007-bib-0087] Osborne, M. J. , S. R. Davenport , C. W. Hoagstrom , and T. F. Turner . 2010. “Genetic Effective Size, ne, Tracks Density in a Small Freshwater Cyprinid, Pecos Bluntnose Shiner (*Notropis Simus Pecosensis*).” Molecular Ecology 19: 2832–2844. 20579288 10.1111/j.1365-294X.2010.04695.x

[eva70007-bib-0088] Pannell, J. R. 2015. “Evolution of the Mating System in Colonizing Plants.” Molecular Ecology 24: 2018–2037. 25611580 10.1111/mec.13087

[eva70007-bib-0089] Parana, J. D. , J. D. Parana , E. Federico , and C. C. Jr . 2022. “Sex‐Reversal of Male Freshwater Prawn (*Macrobrachium Dacqueti*) to Neofemale Through Gonadal Ablation.” Journal of Natural and Allied Sciences VI: 8–15.

[eva70007-bib-0090] Pearse, D. E. , and E. C. Anderson . 2009. “Multiple Paternity Increases Effective Population Size.” Molecular Ecology 18: 3124–3127. 19555411 10.1111/j.1365-294X.2009.04268.x

[eva70007-bib-0091] Penn, G. H. 1943. “A Study of the Life History of the Louisiana Red‐Crawfish, *Cambarus Clarkii* Girard.” Ecology 24: 1–18.

[eva70007-bib-0092] Prakash, S. S. , M. M. Lal , P. H. Dutton , C. Rico , and S. Piovano . 2022. “Kinship Genomics Approach to Study Mating Systems in a Depleted Sea Turtle Rookery.” Regional Studies in Marine Science 51: 102174.

[eva70007-bib-0093] Prystupa, S. , G. R. McCracken , R. Perry , and D. E. Ruzzante . 2021. “Population Abundance in Arctic Grayling Using Genetics and Close‐Kin Mark‐Recapture.” Ecology and Evolution 11: 4763–4773. 33976846 10.1002/ece3.7378 PMC8093667

[eva70007-bib-0094] Pyšek, P. , and D. M. Richardson . 2010. “Invasive Species.” Environmental Change and Management, and Health 35: 25–55.

[eva70007-bib-0095] Queffelec, J. , J. D. Allison , J. M. Greeff , and B. Slippers . 2021. “Influence of Reproductive Biology on Establishment Capacity in Introduced Hymenoptera Species.” Biological Invasions 23: 387–406.

[eva70007-bib-0096] Quinlan, A. R. , and I. M. Hall . 2010. “BEDTools: A Flexible Suite of Utilities for Comparing Genomic Features.” Bioinformatics 26: 841–842. 20110278 10.1093/bioinformatics/btq033 PMC2832824

[eva70007-bib-0097] Rafajlović, M. , A. Eriksson , A. Rimark , et al. 2013. “The Effect of Multiple Paternity on Genetic Diversity of Small Populations During and After Colonisation.” PLoS One 8: e75587. 24204577 10.1371/journal.pone.0075587 PMC3810386

[eva70007-bib-0098] Ramalho, R. O. , and P. M. Anastácio . 2015. “Factors Inducing Overland Movement of Invasive Crayfish (*Procambarus clarkii*) in a Ricefield Habitat.” Hydrobiologia 746: 135–146.

[eva70007-bib-0099] Rochette, N. C. , A. G. Rivera‐Colón , and J. M. Catchen . 2019. “Stacks 2: Analytical Methods for Paired‐End Sequencing Improve RADseq‐Based Population Genomics.” Molecular Ecology 28: 4737–4754. 31550391 10.1111/mec.15253

[eva70007-bib-0100] Rodríguez, C. F. , E. Bécares , and M. Fernández‐Aláez . 2003. “Shift From Clear to Turbid Phase in Lake Chozas (NW Spain) due to the Introduction of American Red Swamp Crayfish (*Procambarus clarkii*).” Hydrobiologia 506: 421–426.

[eva70007-bib-0101] Rohland, N. , and D. Reich . 2012. “Cost‐Effective, High‐Throughput DNA Sequencing Libraries for Multiplexed Target Capture.” Genome Research 22: 939–946. 22267522 10.1101/gr.128124.111 PMC3337438

[eva70007-bib-0102] Rungsin, W. , N. Paankhao , and U. Na‐Nakorn . 2006. “Production of all‐Male Stock by Neofemale Technology of the Thai Strain of Freshwater Prawn, *Macrobrachium Rosenbergii* .” Aquaculture 259: 88–94.

[eva70007-bib-0103] Ruzzante, D. E. , G. R. McCracken , B. Førland , et al. 2019. “Validation of Close‐Kin Mark‐Recapture (CKMR) Methods for Estimating Population Abundance.” Methods in Ecology and Evolution 10: 1445–1453.

[eva70007-bib-0104] Sakai, A. K. , F. W. Allendorf , J. S. Holt , et al. 2001. “The Population Biology of Invasive Species.” Annual Review of Ecology, Evolution, and Systematics 32: 305–332.

[eva70007-bib-0105] Sard, N. M. , R. D. Hunter , E. F. Roseman , D. B. Hayes , R. L. DeBruyne , and K. T. Scribner . 2021. “Pedigree Accumulation Analysis: Combining Methods From Community Ecology and Population Genetics for Breeding Adult Estimation.” Methods in Ecology and Evolution 12: 13704.

[eva70007-bib-0106] Sard, N. M. , K. R. Smith , B. M. Roth , L. R. Nathan , S. J. Herbst , and K. T. Scribner . 2023. “Multiple Sources Implicated in the Red Swamp Crayfish Invasion in Michigan, USA.” Biological Invasions 25: 1–12.

[eva70007-bib-0107] Sard, N. M. , S. R. Smith , J. J. Homola , et al. 2020. “RAPTURE (RAD Capture) Panel Facilitates Analyses Characterizing Sea Lamprey Reproductive Ecology and Movement Dynamics.” Ecology and Evolution 10: 1469–1488. 32076528 10.1002/ece3.6001 PMC7029094

[eva70007-bib-0108] Schmidt, T. L. , S. Elfekih , L. J. Cao , et al. 2023. “Close kin Dyads Indicate Intergenerational Dispersal and Barriers.” American Naturalist 201: 65–77. 10.1086/722175 36524932

[eva70007-bib-0109] Scholtz, G. , A. Braband , L. Tolley , et al. 2002. “Parthenogenesis in an Outsider Crayfish.” Nature 421: 806. 10.1038/421806a 12594502

[eva70007-bib-0110] Schwartz, M. K. , G. Luikart , and R. S. Waples . 2006. “Genetic Monitoring as a Promising Tool for Conservation and Management.” Trends in Ecology & Evolution 22: 25–33. 16962204 10.1016/j.tree.2006.08.009

[eva70007-bib-0111] Scribner, K. T. , G. Uhrig , J. Kanefsky , et al. 2022. “Pedigree‐Based Decadal Estimates of Lake Sturgeon Adult Spawning Numbers and Genetic Diversity of Stream‐Side Hatchery Produced Offspring.” Journal of Great Lakes Research 48: 551–564.

[eva70007-bib-0112] Seebens, H. , T. M. Blackburn , E. E. Dyer , et al. 2018. “Global Rise in Emerging Alien Species Results From Increased Accessibility of New Source Pools.” Proceedings of the National Academy of Sciences 115: E2264–E2273. 10.1073/pnas.1719429115 PMC5877962 29432147

[eva70007-bib-0113] Seebens, H. , T. M. Blackburn , E. E. Dyer , et al. 2017. “No Saturation in the Accumulation of Alien Species Worldwide.” Nature Communications 8: 14435. 10.1038/ncomms14435 PMC5316856 28198420

[eva70007-bib-0114] Sharma, Y. , J. B. Bennett , G. Rašić , and J. M. Marshall . 2022. “Close‐Kin Mark‐Recapture Methods to Estimate Demographic Parameters of Mosquitoes.” PLoS Computational Biology 18: e1010755. 36508463 10.1371/journal.pcbi.1010755 PMC9779664

[eva70007-bib-0115] Slatyer, R. A. , B. S. Mautz , P. R. Y. Backwell , and M. D. Jennions . 2012. “Estimating Genetic Benefits of Polyandry From Experimental Studies: A Meta‐Analysis.” Biological Reviews 87: 1–33. 21545390 10.1111/j.1469-185X.2011.00182.x

[eva70007-bib-0116] Smerud, J. , J. Rivera , T. Johnson , et al. 2022. Field Application of Carbon Dioxide as a Behavioral Control Method for Invasive Red Swamp Crayfish (Procambarus clarkii) in Southeastern Michigan Water Retention Ponds. U.S. Geological Survey Open‐File Report 2022‐1105, 12 p.

[eva70007-bib-0117] Smith, K. , B. M. Roth , S. J. Herbst , et al. 2018. “Assessment of Invasion Risks for Red Swamp Crayfish (*Procambarus clarkii*) in Michigan, USA.” Management of Biological Invasions 9: 405–415.

[eva70007-bib-0118] Sommer, T. R. 1984. “The Biological Response of the Crayfish *Procambarus clarkii* to Transplantation Into California Ricefields.” Aquaculture 41: 373–384.

[eva70007-bib-0119] Souty‐Grosset, C. , P. M. Anastácio , L. Aquiloni , et al. 2016. “The Red Swamp Crayfish *Procambarus clarkii* in Europe: Impacts on Aquatic Ecosystems and Human Well‐Being.” Limnologica 58: 78–93.

[eva70007-bib-0120] Steele, C. A. , M. Hess , S. Narum , and M. Campbell . 2019. “Parentage‐Based Tagging: Reviewing the Implementation of a New Tool for an old Problem.” Fisheries (Bethesda) 44: 412–422.

[eva70007-bib-0121] Sugg, D. W. , and R. K. Chesser . 1994. “Effective Population Sizes With Multiple Paternity.” Genetics 137: 1147–1155. 7982568 10.1093/genetics/137.4.1147 PMC1206061

[eva70007-bib-0122] Suko, T. 1956. “Studies on the Development of the Crayfish. IV. The Development of Winter Eggs.” Science Reports of the Saitama University: Series B 2: 213–219.

[eva70007-bib-0123] Suko, T. 1958. “Studies on the Development of the Crayfish. VI. The Reproductive Cycle.” Science Reports of the Saitama University: Series B 3: 79–91.

[eva70007-bib-0124] Tallmon, D. A. , D. Gregovich , R. S. Waples , et al. 2010. “When Are Genetic Methods Useful for Estimating Contemporary Abundance and Detecting Population Trends?” Molecular Ecology Resources 10: 684–692. 21565073 10.1111/j.1755-0998.2010.02831.x

[eva70007-bib-0125] Tam, C. K. , W. M. Daniel , E. Campbell , J. J. English , and S. C. Soileau . 2021. “U.S. Geological Survey invasive species research—Improving detection, awareness, decision support, and control (ver. 1.1, May 2022).” U.S. Geological Survey Circular 1485, 28 p.

[eva70007-bib-0126] Taylor, A. T. , M. R. Bangs , and J. M. Long . 2021. “Sibship Reconstruction With SNPs Illuminates the Scope of a Cryptic Invasion of Asian Swamp Eels (*Monopterus Albus*) in Georgia, USA.” Biological Invasions 23: 569–580.

[eva70007-bib-0127] Taylor, M. L. , T. A. R. Price , and N. Wedell . 2014. “Polyandry in Nature: A Global Analysis.” Trends in Ecology & Evolution 28: 376–383. 10.1016/j.tree.2014.04.005 24831458

[eva70007-bib-0128] Tricarico, E. , L. Vilizzi , F. Gherardi , and G. H. Copp . 2010. “Calibration of FI‐ISK, an Invasiveness Screening Tool for Nonnative Freshwater Invertebrates.” Risk Analysis 30: 285–292. 19572968 10.1111/j.1539-6924.2009.01255.x

[eva70007-bib-0129] Twardochleb, L. A. , J. D. Olden , and E. R. Larson . 2013. “A Global Meta‐Analysis of the Ecological Impacts of Nonnative Crayfish.” Freshwater Science 32: 1367–1382.

[eva70007-bib-0130] Venables, W. N. , and B. D. Ripley . 2002. Modern Applied Statistics With S. Fourth ed. New York, NY: Springer.

[eva70007-bib-0131] Verhaegen, G. , K. von Jungmeister , and M. Haase . 2021. “Life History Variation in Space and Time: Environmental and Seasonal Responses of a Parthenogenetic Invasive Freshwater Snail in Northern Germany.” Hydrobiologia 848: 2153–2168.

[eva70007-bib-0132] Vogt, G. 2012. “Ageing and Longevity in the Decapoda (Crustacea): A Review.” Zoologischer Anzeiger 251: 1–25.

[eva70007-bib-0133] Vu, N. T. T. , D. R. Jerry , R. C. Edmunds , D. B. Jones , and K. R. Zenger . 2023. “Development of a Global SNP Resource for Diversity, Provenance, and Parentage Analyses on the Indo‐Pacific Giant Black Tiger Shrimp (*Penaeus Monodon*).” Aquaculture 563: 738890.

[eva70007-bib-0134] Walker, D. , B. A. Porter , and J. C. Avise . 2002. “Genetic Parentage Assessment in the Crayfish *Orconectes Placidus*, a High‐Fecundity Invertebrate With Extended Maternal Brood Care.” Molecular Ecology 11: 2115–2122. 12296953 10.1046/j.1365-294x.2002.01609.x

[eva70007-bib-0135] Wang, J. 2004. “Sibship Reconstruction From Genetic Data With Typing Errors.” Genetics 166: 1963–1979. 15126412 10.1534/genetics.166.4.1963 PMC1470831

[eva70007-bib-0136] Wang, J. 2009. “A New Method for Estimating Effective Population Sizes From a Single Sample of Multilocus Genotypes.” Molecular Ecology 18: 2148–2164. 19389175 10.1111/j.1365-294X.2009.04175.x

[eva70007-bib-0137] Wang, J. 2016. “A Comparison of Single‐Sample Estimators of Effective Population Sizes From Genetic Marker Data.” Molecular Ecology 25: 4692–4711. 27288989 10.1111/mec.13725

[eva70007-bib-0138] Wang, J. , and A. W. Santure . 2009. “Parentage and Sibship Inference From Multilocus Genotype Data Under Polygamy.” Genetics 181: 1579–1594. 19221199 10.1534/genetics.108.100214 PMC2666522

[eva70007-bib-0139] Waples, R. K. , W. A. Larson , and R. S. Waples . 2016. “Estimating Contemporary Effective Population Size in Non‐model Species Using Linkage Disequilibrium Across Thousands of Loci.” Heredity 117: 233–240. 27553452 10.1038/hdy.2016.60 PMC5026758

[eva70007-bib-0140] Waples, R. S. , T. Antao , and G. Luikart . 2014. “Effects of Overlapping Generations on Linkage Disequilibrium Estimates of Effective Population Size.” Genetics 197: 769–780. 24717176 10.1534/genetics.114.164822 PMC4063931

[eva70007-bib-0141] Waples, R. S. , and C. Do . 2010. “Linkage Disequilibrium Estimates of Contemporary *N* _e_ Using Highly Variable Genetic Markers: A Largely Untapped Resource for Applied Conservation and Evolution.” Evolutionary Applications 3: 244–262. 25567922 10.1111/j.1752-4571.2009.00104.x PMC3352464

[eva70007-bib-0142] Weise, E. M. , K. T. Scribner , J. V. Adams , et al. 2022. “Pedigree Analysis and Estimates of Effective Breeding Size Characterize Sea Lamprey Reproductive Biology.” Evolutionary Applications 15: 484–500. 35386399 10.1111/eva.13364 PMC8965388

[eva70007-bib-0143] Weise, E. M. , K. T. Scribner , O. Boeberitz , G. Bravener , N. S. Johnson , and J. D. Robinson . 2023. “Evaluating the Utility of Effective Breeding Size Estimates for Monitoring Sea Lamprey Spawning Abundance.” Ecology and Evolution 13: e10519. 37745785 10.1002/ece3.10519 PMC10511834

[eva70007-bib-0144] Williams, B. W. , D. R. Etter , D. W. Linden , K. F. Millenbah , S. R. Winterstein , and K. T. Scribner . 2009. “Noninvasive Hair Sampling and Genetic Tagging of Co‐Distributed Fishers and American Martens.” Journal of Wildlife Management 73: 26–34.

[eva70007-bib-0145] Wright, S. 1931. “Evolution in Mendelian Populations.” Genetics 16: 97–159. 17246615 10.1093/genetics/16.2.97 PMC1201091

[eva70007-bib-0146] Wu, N. , H. Wei , H. H. Shen , T. T. Wu , and M. Guo . 2012. “Acute Toxic Effects of Deltamethrin on Red Swamp Crayfish, *Procambarus clarkii* (Decapoda, Cambaridae).” Crustaceana 85: 993–1005.

[eva70007-bib-0147] Xu, Z. , T. Gao , Y. Xu , et al. 2021. “A Chromosome‐Level Reference Genome of Red Swamp Crayfish *Procambarus clarkii* Provides Insights Into the Gene Families Regarding Growth or Development in Crustaceans.” Genomics 113: 3274–3284. 34303807 10.1016/j.ygeno.2021.07.017

[eva70007-bib-0148] Yue, G. H. , J. Le Li , C. M. Wang , J. H. Xia , G. L. Wang , and J. B. Feng . 2010. “High Prevalence of Multiple Paternity in the Invasive Crayfish Species, *Procambarus clarkii* .” International Journal of Biological Sciences 6: 107. 20186292 10.7150/ijbs.6.107 PMC2828620

[eva70007-bib-0149] Yue, G. H. , G. L. Wang , B. Q. Zhu , C. M. Wang , Z. Y. Zhu , and L. C. Lo . 2008. “Discovery of Four Natural Clones in a Crayfish Species *Procambarus clarkii* .” International Journal of Biological Sciences 4: 279. 18781225 10.7150/ijbs.4.279 PMC2532795

[eva70007-bib-0150] Zalewski, A. , H. Zalewska , S.‐G. Lunneryd , C. André , and G. Mikusiński . 2016. “Reduced Genetic Diversity and Increased Structure in American Mink on the Swedish Coast Following Invasive Species Control.” PLoS One 11: e0157972. 27333328 10.1371/journal.pone.0157972 PMC4917106

[eva70007-bib-0151] Zeng, Y. , D. Díez‐del‐Molino , O. Vidal , M. Vera , and J. L. García‐Marín . 2017. “Multiple Paternity and Reproduction Opportunities for Invasive Mosquitofish.” Hydrobiologia 795: 139–151.

