# Supplementary material for: Genomic Data Characterize Reproductive Ecology Patterns in Michigan Invasive Red Swamp Crayfish (Procambarus clarkii)
Source: Evol Appl. 2024 Sep 15;17(9):e70007. doi: 10.1111/eva.70007 (PMC11403126; doi:10.1111/eva.70007)
Supplement: Supplementary file 1 — Appendix S1. [file EVA-17-e70007-s001.zip › 3_RSC_ms1_suppMeths_9-7-24.docx]

**Genomic data characterize reproductive ecology patterns in Michigan invasive Red Swamp Crayfish (*Procambarus clarkii*)**

Nicole E. Adams^1^, Jared J. Homola^2^, Nicholas M. Sard^3^, Lucas R. Nathan^4^, Brian M. Roth^1^, John D. Robinson^1^, Kim T. Scribner^1^

^1^ Department of Fisheries and Wildlife, Michigan State University, Natural Resources Building, 480 Wilson Rd., East Lansing, MI 48824, USA

^2^ U. S. Geological Survey, Wisconsin Cooperative Fishery Research Unit, College of Natural Resources, University of Wisconsin-Stevens Point, Stevens Point, WI 54481, USA

^3^ Biological Sciences Department, The State University of New York – Oswego, 7060 State Route 104, Oswego, NY 13126, USA

^4^ Michigan Department of Natural Resources, 525 W. Allegan St, Lansing, MI 48933, USA

**Supplementary methods**: RAD capture bait panel design

*RAD-seq library for locus discovery*

We generated a RAD capture SNP genotyping panel (Ali et al. 2016) for genetic monitoring of invasive Red Swamp Crayfish (*Procambarus clarkii*) populations. Restriction site-associated DNA (RAD) sequencing was used to previously generate a ‘discovery’ RADseq library (Sard et al. 2023) that allowed us to identify loci for use in RAD capture bait design. The discovery dataset was generated from 175 Red Swamp Crayfish collected from 13 locations in the species’ native and invaded ranges (see Table 1 in Sard et al. (2023) for details). Gill tissues from each individual were preserved in ethanol. Genomic DNA was extracted using a spin column-based approach (DNeasy Blood and Tissue kits; Qiagen, Hilden, Germany) following the manufacturer's protocols.

The discovery RAD library was prepared following the “BestRAD” protocol (Ali et al. 2016). Briefly, genomic DNA was digested with *SbfI* restriction enzyme (New England Biolabs, Ipswich, MA, USA). BestRAD adapters (New England Biolabs) were ligated to the cut ends using T4 Ligase (New England Biolabs). Samples were pooled and then sheared with a sonicator (Covaris M220; Woburn, MA, USA). Barcoded, sheared DNA were isolated with Dynabeads M-280 streptavidin beads (Invitrogen, Waltham, MA, USA) then libraries were prepared following NEBNext Library prep kit (New England Biolabs). Libraries were dual-indexed using NEB Dual Index Sets 1 and amplified for 12 cycles followed by a magnetic bead-based cleanup (Ampure XP bead; Beckman Coulter, Brea, CA, USA).

The discovery RAD library was sequenced by collecting 150-bp reads on three HiSeq 4000 lanes at Novogene (Sacramento, CA). Sequence reads were processed as described in Sard et al. (2023) using Stacks v. 1.44 (Catchen et al. 2011, 2013). Libraries were demultiplexed using process_radtags and PCR duplicates were removed using clone_filter. Because the Red Swamp Crayfish genome (GCA_020424385.2; Xu et al. 2021) was not yet available, we mapped reads to the Marbled Crayfish (*Procambarus virginalis*) reference genome (NCBI Bioproject accession number PRJNA356499; (Gutekunst et al. 2018)) using BWA-MEM (Li and Durbin 2010; Li 2013). Genotypes were then called using the gstacks module of Stacks v. 1.44 (Catchen et al. 2013; Rochette et al. 2019). Once the *P. clarkii* genome was available, we aligned the baits to it using BWA-MEM to evaluate mapping rates and the genomic location of each bait.

*RAD capture locus selection*

The polymorphic SNP loci identified in the discovery library provided a set of candidate loci for designing capture baits to perform RAD capture genotyping (Ali et al. 2016). To begin the process of locus selection, we first removed samples with low confidence genotype calls (genotype quality score <20). Next, we removed SNPs with >50% missing data. SNPs with a minor allele count less than three, and those with an average read count across samples of less than six. We then filtered on heterozygosity by removing SNPs without heterozygotes and those with observed heterozygosity greater than 0.6. We then again filtered on the amount of missing data by removing SNPs with a missing data frequency greater than 10%. Next, we removed SNPs that occurred in RAD loci that had more than five SNPs. SNPs outside the first 140 bp of each RAD locus were then removed, as well as those with allele balance values less than 0.4 or greater than 0.6. Finally, we calculated the mean depth at all remaining SNPs and retained only those with a depth within one standard deviation of the mean depth. Consensus sequences for each RAD locus containing SNPs that passed all filters were then sent to Arbor Biosciences (Ann Arbor, Michigan) for additional quality evaluation to ensure an absence of heterodimers with other baits and less than 25% repeat masking. Finally, three 80 bp tiled baits were designed for each retained RAD locus. A file of RAD baits can be found in the supplement, and with Ref#201001-90 at Arbor Biosciences. Finally, once the *P. clarkii* genome was available, we aligned the baits to it using BWA MEM to evaluate mapping rates and the genomic location of each bait.

**References**

Ali, O. A., S. M. O’Rourke, S. J. Amish, M. H. Meek, G. Luikart, C. Jeffres, and M. R. Miller. 2016. RAD Capture (Rapture): Flexible and Efficient Sequence-Based Genotyping. Genetics 202:389–400.

Catchen, J., P. A. Hohenlohe, S. Bassham, A. Amores, and W. A. Cresko. 2013. Stacks: An Analysis Tool Set for Population Genomics. Mol Ecol 22:3124–3140.

Catchen, J. M., A. Amores, P. Hohenlohe, W. Cresko, and J. H. Postlethwait. 2011. Stacks: Building and Genotyping Loci De Novo from Short-Read Sequences. G3 Genes|Genomes|Genetics 1:171–182.

Gutekunst, J., R. Andriantsoa, C. Falckenhayn, K. Hanna, W. Stein, J. Rasamy, and F. Lyko. 2018. Clonal Genome Evolution and Rapid Invasive Spread of the Marbled Crayfish. Nat Ecol Evol 2:567–573.

Li, H. 2013. Aligning Sequence Reads, Clone Sequences and Assembly Contigs with BWA-MEM. ArXiv 1303.3997 [q-bio.GN].

Li, H., and R. Durbin. 2010. Fast and Accurate Long-read Alignment with Burrows-Wheeler Transform. Bioinformatics 26:589–95.

Rochette, N. C., A. G. Rivera-Colón, and J. M. Catchen. 2019. Stacks 2: Analytical Methods for Paired-end Sequencing Improve RADseq-based Population Genomics. Mol Ecol 28:4737–4754.

Sard, N. M., K. R. Smith, B. M. Roth, L. R. Nathan, S. J. Herbst, and K. T. Scribner. 2023. Multiple Sources Implicated in the Red Swamp Crayfish Invasion in Michigan, USA. Biol Invasions.

Xu, Z., T. Gao, Y. Xu, X. Li, J. Li, H. Lin, W. Yan, J. Pan, and J. Tang. 2021. A Chromosome-Level Reference Genome of Red Swamp Crayfish *Procambarus clarkii* Provides Insights into the Gene Families Regarding Growth or Development in Crustaceans. Genomics 113:3274–3284.
