# Supplementary material for: Genomic Data Characterize Reproductive Ecology Patterns in Michigan Invasive Red Swamp Crayfish (Procambarus clarkii)
Source: Evol Appl. 2024 Sep 15;17(9):e70007. doi: 10.1111/eva.70007 (PMC11403126; doi:10.1111/eva.70007)
Supplement: Supplementary file 1 — Appendix S1. [file EVA-17-e70007-s001.zip › 6_Supplement_TrackChanges_9-7-24.docx]

*Supplementary tables and figures*

Table S1. Summary of sample sizes of *Procambarus clarkii* used for analyses from the following sites: East Golf Course 1 (GC1), East Golf Course 1 (GC2), East Golf Course 4 (GC4), West Golf Course 1 (GC1), and Hotel1. Dashes indicate no data collected.

| Waterbody | Year collected | Juveniles | Berried females | Eggs/ hatchlings | Adult males |
| --- | --- | --- | --- | --- | --- |
| EastGC1 | 2020 | — | — | — | 5 |
|  | 2021 | — | 5 | 152 | — |
| EastGC2 | 2020 | 67 | 1 | 34 | 20 |
|  | 2021 | 51 | — | — | 22 |
| EastGC4 | 2020 | 86 | 3 | 76 | 21 |
|  | 2021 | 208 | 1 | 35 | 20 |
| WestGC1 | 2020 | 64 | 1 | 33 | 17 |
|  | 2021 | 185 | 5 | 176 | 40 |
| Hotel1 | 2020 | 81 | 5 | 140 | 22 |
|  | 2021 | 160 | 1 | 18 | 45 |

Table S2. Details for catch per unit effort (CPUE) for *Procambarus clarkii* and site size estimates across five waterbodies from 2019-2021. The dash indicates CPUE was not calculated.

| Waterbody | Abbreviation | Year | Nsurveys | Total traps | August CPUE | Perimeter (m) | Acres |
| --- | --- | --- | --- | --- | --- | --- | --- |
| East Golf Course 1 | EastGC1 | 2019 | 71 | 3629 | 3.637 | 334 | 0.43 |
| East Golf Course 1 | EastGC1 | 2020 | 65 | 2785 | 3.181 |  |  |
| East Golf Course 1 | EastGC1 | 2021 | 81 | 3509 | 2.839 |  |  |
| East Golf Course 2 | EastGC2 | 2019 | 72 | 2152 | 1.982 | 254 | 0.72 |
| East Golf Course 2 | EastGC2 | 2020 | 21 | 690 | 1.453 |  |  |
| East Golf Course 2 | EastGC2 | 2021 | 35 | 1282 | — |  |  |
| East Golf Course 4 | EastGC4 | 2019 | 56 | 2452 | 2.092 | 432 | 0.60 |
| East Golf Course 4 | EastGC4 | 2020 | 55 | 2713 | 2.024 |  |  |
| East Golf Course 4 | EastGC4 | 2021 | 82 | 3966 | 0.296 |  |  |
| West Golf Course 1 | WestGC1 | 2019 | 64 | 1620 | 1.917 | 151 | 0.36 |
| West Golf Course 1 | WestGC1 | 2020 | 69 | 1962 | 5.453 |  |  |
| West Golf Course 1 | WestGC1 | 2021 | 93 | 2444 | 2.896 |  |  |
| Hotel1 | Hotel1 | 2019 | 89 | 3953 | 0.723 | 280 | 0.95 |
| Hotel1 | Hotel1 | 2020 | 71 | 3144 | 3.752 |  |  |
| Hotel1 | Hotel1 | 2021 | 94 | 3729 | 0.027 |  |  |

Table S3. Inferred parents contributing to juveniles in multiple cohorts of *Procambarus clarkii* at the Hotel1 site. Cohort 1 is defined as samples collected during Fall 2020 to Spring 2021 and samples in cohort 2 were collected during Summer 2021 to Fall 2021.

| Parent 1 ID | Number of juveniles in Cohort 1 | Cohort 1  Parent 2 ID | Number of juveniles in Cohort 2 | Cohort 2  Parent 2 ID | Halfsibling probability |
| --- | --- | --- | --- | --- | --- |
| #8 | 1 | *9 | 1 | *21 | 0.85 |
| #13 | 1 | *27 | 4 | *12, *15 (N=2), *21 | 0.85 |
| #19 | 1 | *18 | 1 | *34 | 0.36 |
| #24 | 2 | *25, *28 | 1 | *40 | 0.14 |
| #28 | 1 | *31 | 1 | *35 | 0.26 |
| #31 | 6 | *24 (N=6) | 5 | *35 (N=5) | 0.26 |
| *17 | 1 | #32 | 4 | #18 (N=4) | 0.27 |
| *18 | 2 | #25, #27 | 1 | #19 | 0.36 |
| *21 | 1 | #8 | 1 | #13 | 0.85 |
| *25 | 7 | #22 (N=6), #24 | 1 | #35 | 0.14 |

Table S5. Results from the generalized linear model selection to understand factors affecting the number of inferred mates. The best model based on the lowest Akaike information criterion (AIC) value is listed first, followed by the remaining four models with their respective difference in AIC value from the best model (ΔAIC). Details are provided only for the best model and significant terms are shown in bold text. The global model used (5) included the following variables: the number of offspring genotyped per berried female, waterbody, average August site-specific catch per unit effort (CPUE; estimate of population density), and the years the berried females (and offspring) were collected.

| Model | Estimate | STDE* | Statistic | P-value | AIC | ΔAIC |
| --- | --- | --- | --- | --- | --- | --- |
| 1) Inferred mates ~ Offspring sample size + CPUE + Year | | | | | 74.87 | — |
| Intercept | 4601.50 | 1392.89 | 3.30 | **0.004** |  |  |
| Offspring sample size | 0.09 | 0.07 | 1.36 | 0.194 |  |  |
| CPUE | -0.83 | 0.34 | -2.41 | **0.028** |  |  |
| Year | -2.276 | 0.689 | -3.301 | **0.005** |  |  |
|  |  |  |  |  |  |  |
| 2) Inferred mates ~ Offspring sample size + CPUE + Year + Carapace length | | | | | 76.84 | 1.97 |
| 3) Inferred mates ~ Offspring sample size + CPUE + Year + Carapace length + Waterbody | | | | | 81.36 | 6.49 |

* STDE, standard error


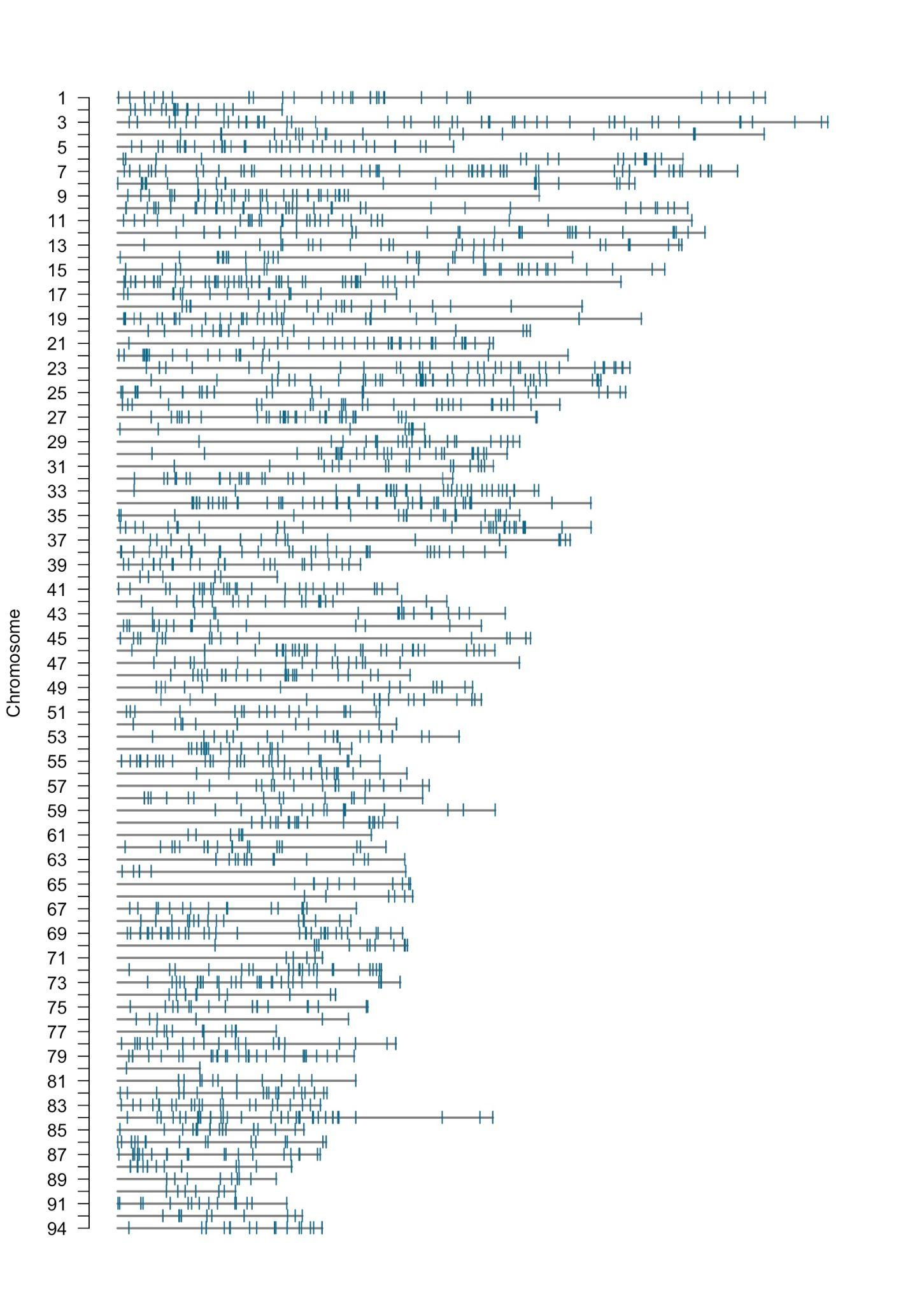

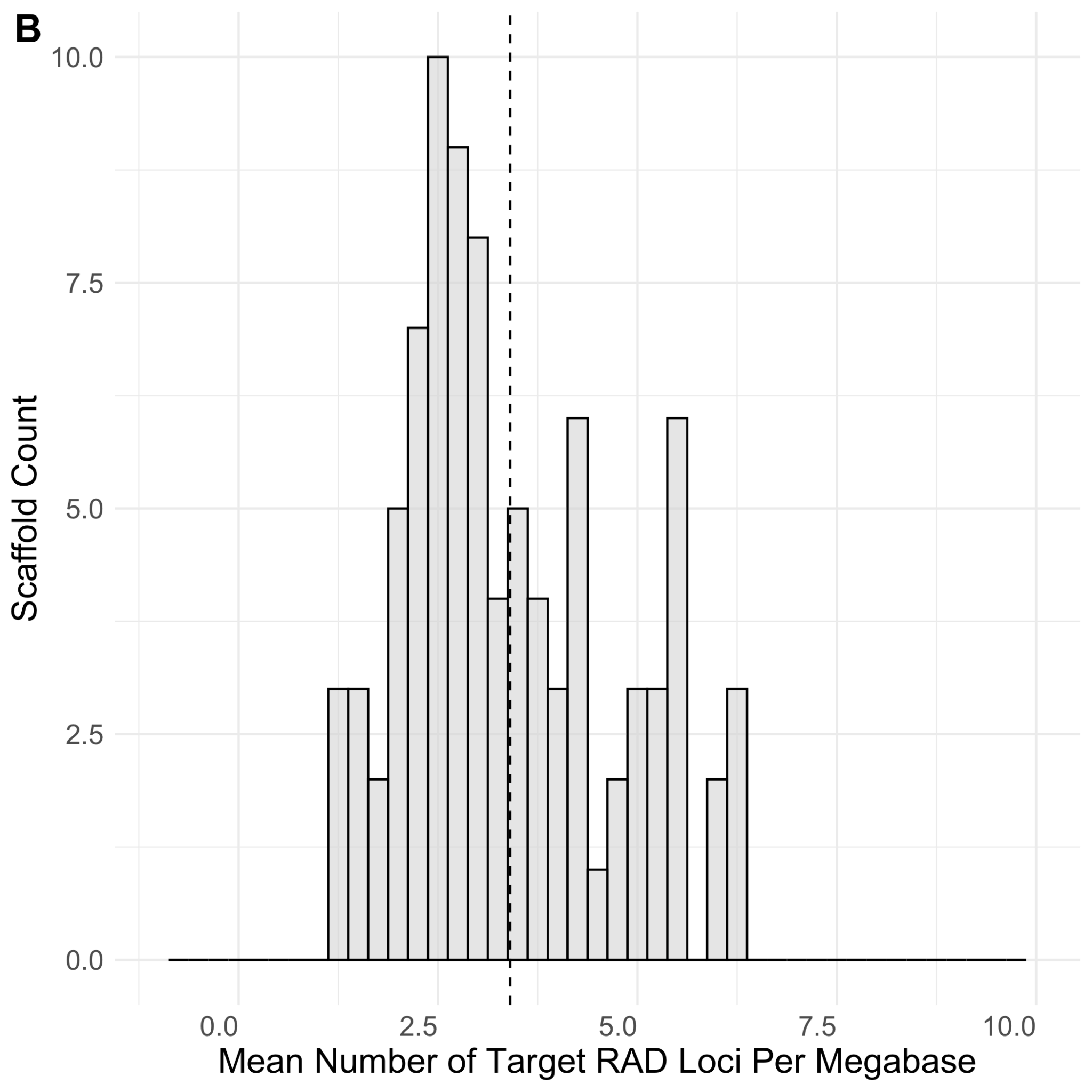


Figure S1. A) Distribution of target RAD loci on 94 *P. clarkii* chromosomes. Each horizontal black line represents a putative chromosome (scaffold) and each vertical line represents the location of a targeted SNP locus. B) The distribution of the density of targeted loci that were greater than 10MB in length (N=89). The dashed vertical line is the average density which is 3.4 RAD loci per megabase (SD = 1.3).


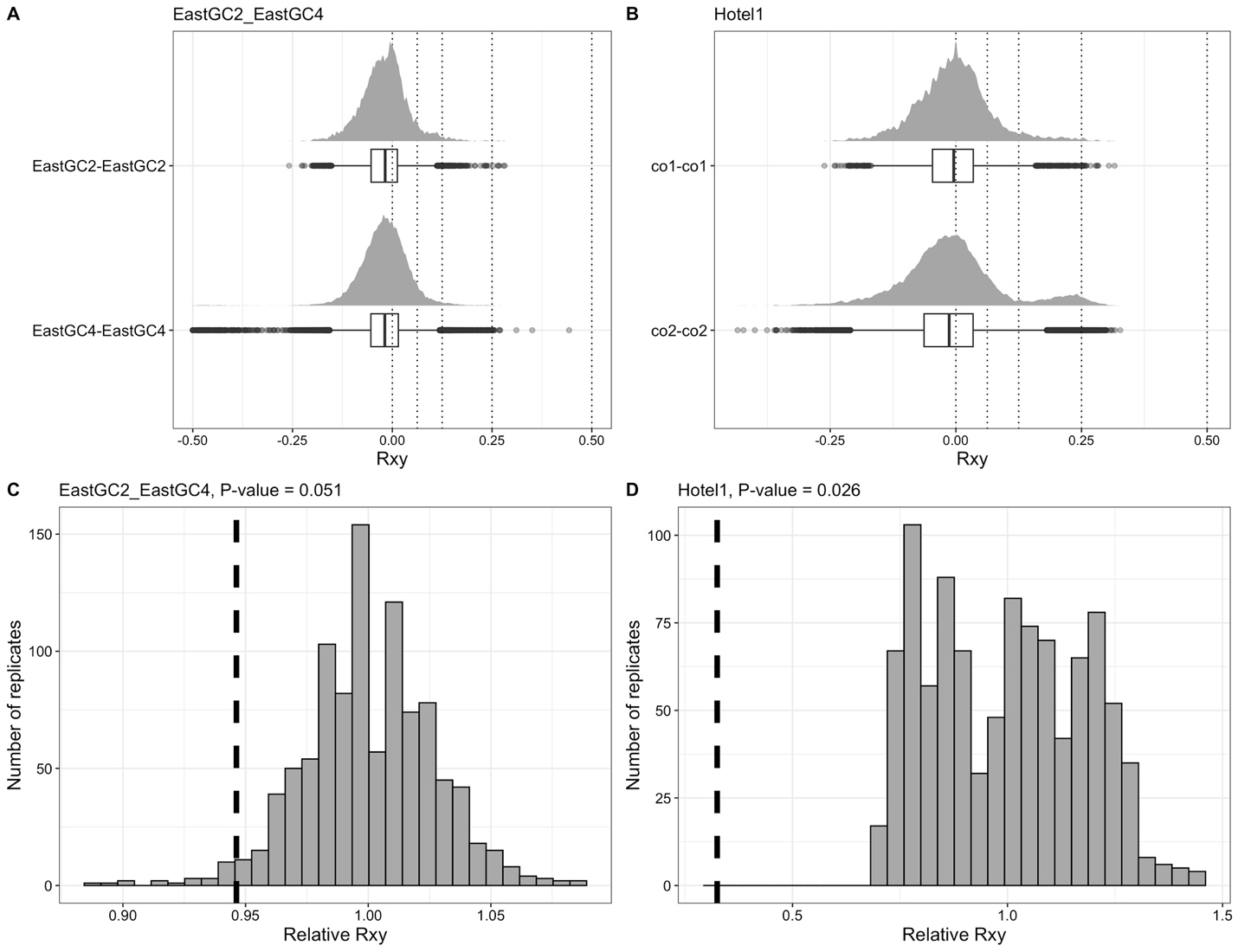


Figure S2. A-B) Estimates of the pairwise relatedness, based on the KING inference in Manichaikul et al. (2010) implemented in VCFtools, of Red Swamp Crayfish (*P. clarkii*) juveniles in East Golf Course (GC) 2, East Golf Course 4, and Hotel1 between cohort1 and cohort2. Estimates were based on 930 SNPs. Dotted vertical lines are relationship thresholds, defined left to right as the following: unrelated, 3rd degree, half-sibling, full-sibling or parent-offspring, and monozygotic twins. There were an additional 226 and 1070 pairs in EastGC4 and WestGC1, respectively, with relatedness less than -0.50 that are not shown here for clarity. C-D) Null distributions of the ratios of median Rxy estimates produced by 1000 randomizations. The black vertical line is the ratio of median Rxy from the empirical data.


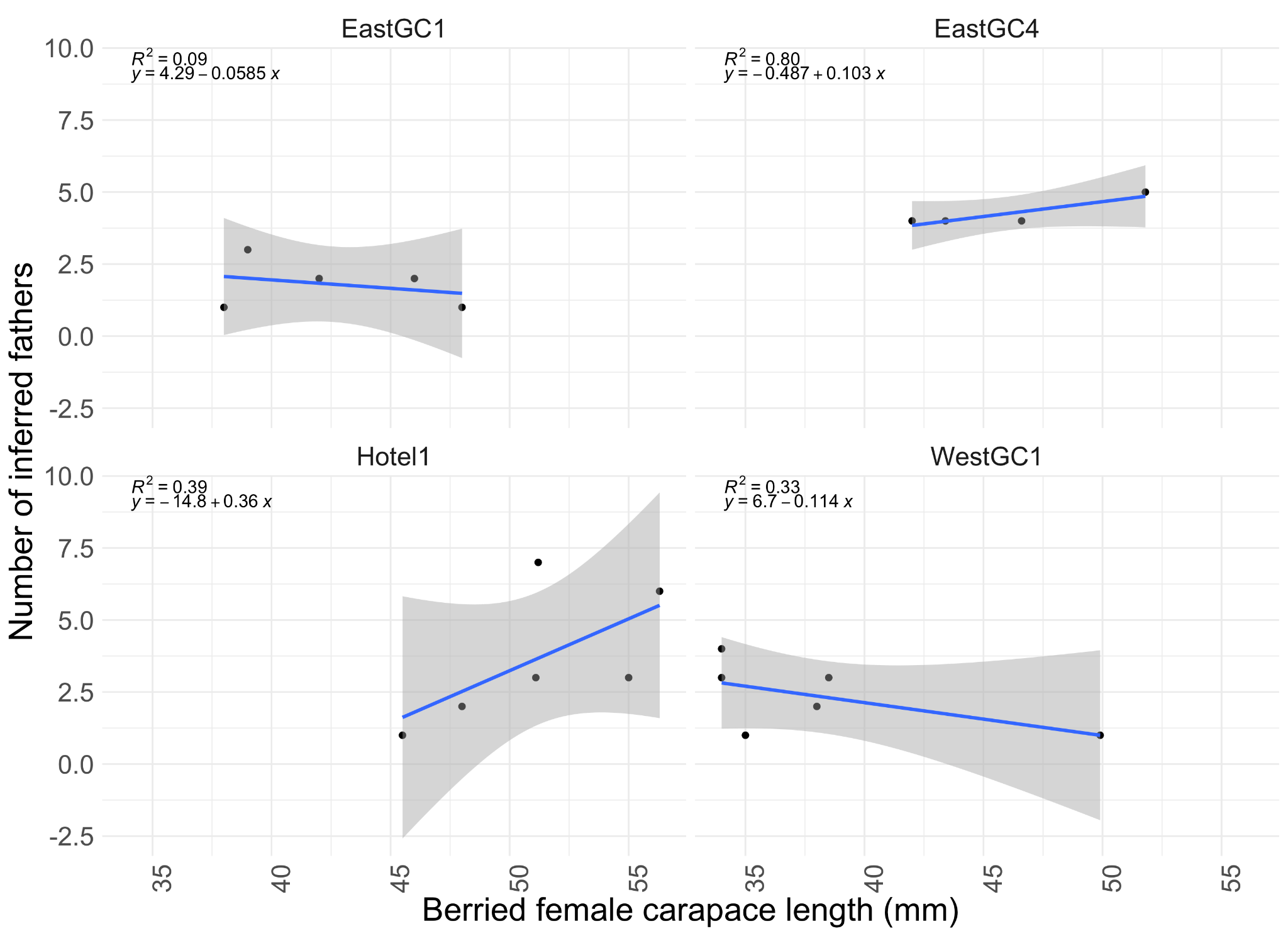


Figure S3. The relationships between the carapace length (mm) of berried females and the number of inferred mates that contributed to their offspring for four waterbodies: East Golf Course (GC) 1, East Golf Course 4, West Golf Course 1, and Hotel1. Linear model equations and R^2^ values are provided for each waterbody. East Golf Course 2 is not shown because it only had one berried female.
